# The puzzle of plant hybridisation: a high propensity to hybridise but few hybrid zones reported

**DOI:** 10.1038/s41437-023-00654-1

**Published:** 2023-10-27

**Authors:** Gonzalo Nieto Feliner, David Criado Ruiz, Inés Álvarez, Irene Villa-Machío

**Affiliations:** Real Jardín Botánico (RJB), CSIC, Plaza de Murillo 2, 28014 Madrid, Spain

**Keywords:** Speciation, Evolutionary genetics

## Abstract

An interesting conundrum was recently revealed by R. Abbott when he found that the number of hybrid zones reported in the literature for plants is very low, given the propensity of plants to hybridise. In another literature survey on hybrid zones performed over the period 1970–2022, we found that the number of hybrid zones reported for vertebrates was 2.3 times greater than that reported for vascular plants, even though there are about six times more vascular plant species than vertebrates. Looking at the number of papers reporting hybrid zones, there are 4.9 times more on vertebrates than on vascular plants. These figures support the relevance of this conundrum. In this paper we aim to shed light on this question by providing a structured discussion of the causes that may underlie this conundrum. We propose six non-mutually exclusive factors, namely lack or deficit of spatial structure, lack or deficit of genetic structure, effects of hybridisation between non-closely related species, lability of plant hybrid zones over time, botanists’ perception of hybridisation, and deficit of population genetic data. There does not appear to be a single factor that explains our puzzle, which applies to all cases of plants where hybridisation is detected but no hybrid zone is reported. It is argued that some plant features suggest that the puzzle is not, at least entirely, due to insufficient knowledge of the specific cases, a hypothesis that should be addressed with a wider range of empirical data across different taxonomic groups.

## Introduction

Natural hybridisation is the crossing of individuals from populations which are distinguishable on the basis of one or more heritable characters (Harrison 1990; Arnold 1997). Along with the massive accumulation of evidence that it is a common phenomenon across the tree of life (Mallet 2005; Folk et al. 2018; Runemark et al. 2019; Edelman and Mallet 2021), genomic-based studies in recent years have also confirmed the diversity and complexity of its consequences (Abbott et al. 2013; Payseur and Rieseberg 2016; Thawornwattana et al. 2022; Bock et al. 2023), which hinder the understanding of its overall evolutionary impact. Several outcomes of natural hybridisation have traditionally been recognised ranging from those in which F1 offspring are consistently sterile—as are mules and many plant hybrids in Stace et al. (2015)—to hybrid speciation. For animal groups, the most commonly reported outcome of hybridisation is the establishment of a situation that persists over time in which two species mate and produce offspring within a defined area known as hybrid zones (HZs hereafter).

Abbott (2017) raised an interesting question when he reported that only 137 hybrid zones were found in plants of equivalent ploidy in a literature search. Kawakami and Butlin (2012) and Hewitt (2004) mentioned that several hundred hybrid zones had been reported in the literature, although they did not provide a list. To put the number of plant hybrid zones in context —without aiming for an exhaustive search— we conducted a survey in Google scholar of papers published in all journals from 1970 to 2022 that included ‘hybrid zone’ in their title. Using this criterion, 371 HZs were identified, of which 86 (23.1%) involved vascular plants and 285 (76.8%) animals (Table 1, S1, S2). The number of vascular plant HZs is similar to or higher than those reported for other large groups such as insects (66; 17.7%) and other invertebrates (18; 4.8%). However, the number of vascular plant HZs is less than half that of another well-studied group such as vertebrates (201; 54%). This contrast is accentuated when looking at the total number of papers —136 for vascular plant HZs vs. 675 for vertebrate HZs— because the latter are often the subject of multiple studies and papers (Table 1). The question that arises from these figures is whether the number of reported HZs for vascular plants should be higher than it is, especially considering that the number of vascular plant species is estimated to be six times that of vertebrates, and the estimated percentage of hybridising species follows the same trend (about 25% for plants vs. about 10% for animals; Mallet 2005). Shedding light on this apparent inconsistency is relevant at a time when the evolutionary consequences of hybridisation in a variety of organisms are being intensely studied along multiple lines of evidence (Green et al. 2010; Abbott et al. 2013; Pease et al. 2016; Vallejo‐Marín and Hiscock 2016; Meier et al. 2017; Lamichhaney et al. 2018; Suarez-Gonzalez et al. 2018; Runemark et al. 2019; Helleu et al. 2022; Hibbins and Hahn 2022). Abbott (2017) suggested that differences in reported plant and animal hybrid zones might not be the result of insufficient sampling in plants, but instead that plant taxa might produce hybrids without forming hybrid zones, which could be thus rare in the wild. This is the starting point for this perspective paper, in which we discuss potential non-mutually exclusive causes that may underlie this puzzle. As the diversity of scenarios and organisms to be considered makes this a challenging task, our aim is to provide a structured discussion that may stimulate studies that address this question with a wide range of empirical data across different taxonomic groups. To address the possible causes of the conundrum, it is important to understand how hybrid zones are defined and recognised.

**Table 1:** Summary from the literature survey presented in Tables S1 and S2 conducted in Google Scholar of papers published in all journals from 1970 to 2022 that included ‘hybrid zone’ in their title (unpublished theses and preprint manuscripts not included).

Group	Nº of HZs	% with respect to all groups	Nº HZs with > 1 paper	% HZs with > 1 paper	Nº papers with HZ in title	AVG Nº papers per HZ	% papers with respect to all groups
VASCULAR PLANTS	86	23.18	20	23.26	136	1.58	16.77
ANIMALS (total)	285	76.82	104	36.49	675	2.37	83.23
Vertebrates	201	54.18	77	38.31	491	2.44	60.54
Insects	66	17.79	23	34.85	154	2.33	18.99
Other invertebrates	18	4.85	4	22.22	30	1.67	3.70
TOTAL	371		124		811		

*HZ*, hybrid zone.

## Hybrid zones

Definitions of hybrid zones tend to be as broad as “narrow regions in which genetically distinct populations meet, mate, and produce hybrids” (Barton and Hewitt 1985) or “narrow regions of phenotypic or genotypic change, which separate otherwise more or less homogeneous taxa” (Jiggins and Mallet 2000). Harrison (1993) even extended the term explicitly to include “situations ranging from sporadic or occasional hybridisation between species that are broadly sympatric (perhaps associated with different habitats or resources) to narrow zones of hybridisation between taxa with effectively parapatric distributions”. This broad circumscription may be useful to fit as many hybridisation scenarios as possible within the HZ framework, but it raises the question of whether classical hybrid zone theory would be useful to investigate the full range of scenarios covered by such broad definitions. In their classic review, Barton and Hewitt (1985) restricted the HZ concept for practical reasons, following the predominant use in the literature, to make it synonymous with a cline, i.e., a gradient or set of gradients in phenotypic characters or allele frequencies, at one or more loci. It is not the purpose of this paper to circumscribe the HZ concept. However, it is relevant to the question we are addressing below, and will therefore be mentioned in the discussion where appropriate.

Hybrid zones have attracted the attention of population geneticists and evolutionary biologists for decades on the grounds that they represent windows into the evolutionary process, providing insights into the genetics of local adaptation, reproductive barriers and speciation (Barton and Hewitt 1985, 1989; Hewitt 1988; Harrison 1990, 1993). More recently, HZs occurring along environmental gradients, have been considered as sentinels for global change whenever climate-driven changes in species ranges can be told apart from other sources of range shifts (Abbott and Brennan 2014; Taylor et al. 2015; Abbott 2017; Wielstra 2019; Abdelaziz et al. 2021).

## Types of hybrid zones

Beyond the production of hybrid offspring, elements that define the nature and types of HZs include the presence of clines, whether hybridisation is restricted to a delimited space, whether this space is environmentally uniform or patchy, whether dispersal is important, whether the scenario is stable over time, and what forces determine its maintenance. Stable hybrid zones can be maintained by selection against hybrids, environmental selection, or a combination of the two (Kawakami and Butlin 2012), and this is essentially what characterises the main types of hybrid zones that have been traditionally considered.

*Tension* models are those in which the HZ is maintained only by a balance between dispersal and selection against hybrids, which are always less fit than the parental taxa (Barton and Hewitt 1985). Since selection is independent of the environment, i.e., endogenous, these HZs are free to move. *Bounded hybrid superiority* zones tend to occur in ecotones between parental habitats, and hybrids exhibit higher fitness than their progenitors only in these intermediate habitats due to environmental-dependent selection, but lower fitness in parental’s habitats (Moore 1977). *Mosaic* zones refer to situations where parental populations occupy distinct habitats that are distributed in a mosaic pattern, and where hybrids may have lower, higher or intermediate fitness than the parental types (Harrison and Rand 1989). To these, Arnold (1997) added the *evolutionary novelty* model, arguing for the involvement of environmental-dependent (exogenous) selection in all the three previous types including the tension model, and for the possibility of a higher fitness in certain hybrid genotypes, not only in ecotones but in certain habitats even in the progenitors’. Unlike other models, in Arnold’s model endogenous selection would not act as a purifying form of selection against all hybrid genotypes but only against some. A rich literature on HZs accumulated over several decades has shown that real situations do not always fit easily into any of these models. This realisation has led to less categorical approaches to HZs such as Curry’s (2015) proposal to place HZ types along a continuum based on selective pressures in different geographical contexts and ignoring criteria such as migration.

Regarding their origin, HZs can be formed in situ by direct environmental selection in contiguous populations across environmental gradients —primary zone— or by secondary contact between previously isolated populations —secondary zone— (Kawakami and Butlin 2012) although it is difficult to distinguish between the two based on current patterns of variation (Endler 1977; Gompert and Buerkle 2016). Another aspect that allows characterisation of HZs is their genetic structure, based on the presence of different genotypic classes. Bimodal hybrid zones are those that contain predominantly multilocus genotypes that are similar to the parental forms, with almost no intermediates (Harrison and Bogdanowicz 1997). Unimodal hybrid zones are those in which intermediate genotypes predominate, i.e., F1s, F2s and backcrosses. Bimodality results from strong prezygotic isolation between the parental populations, whereas unimodal HZs should have weak prezygotic barriers or at least non-assortative mating (Jiggins and Mallet 2000). These authors also suggested that unimodal and bimodal HZs represent different stages of a speciation continuum. This is a useful but controversial idea positing that the different situations of reproductive isolation between diverging species that we currently find in most groups represent different stages of the same speciation process (Shaw and Mullen 2014; Seehausen et al. 2014; Stankowski and Ravinet 2021; DeRaad et al. 2023).

## CAUSES for a low number of plant hybrid zones

**Fig. 1:**
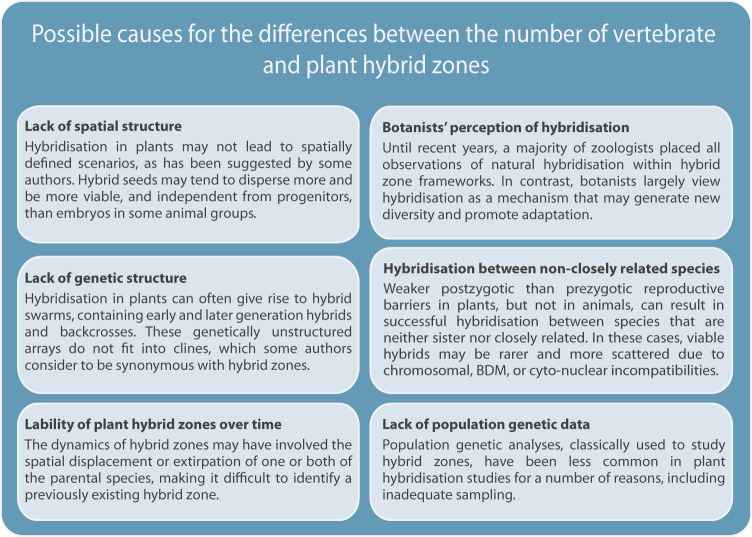
Causes for the few reported hybrid zones in plants compared to vertebrates. Outline of six possible non-mutually exclusive factors discussed in the text.

We propose here six non-mutually exclusive factors that may be involved in this question (Fig. 1). Harrison (1993) stated that “Plant hybrid zones tend to be diffuse (not geographically well defined) and are often characterised by local hybrid swarms”. This statement suggests how the lack or deficit of spatial structure (diffuse HZs) and/or the lack or deficit of genetic structure (hybrid swarms) might be differential features in plant hybridisation scenarios compared to the spatially defined narrow classical HZs in animals. The biological underpinnings of these factors may be a manifestation of the broader range of scenarios along which hybridisation occurs in plant groups, which may be consistent with the propensity of plant species to produce hybrids (Mallet 2005) and the lower strength of postzygotic barriers (Lowry et al. 2008; Widmer et al. 2009; Baack et al. 2015).

**Fig. 2:**
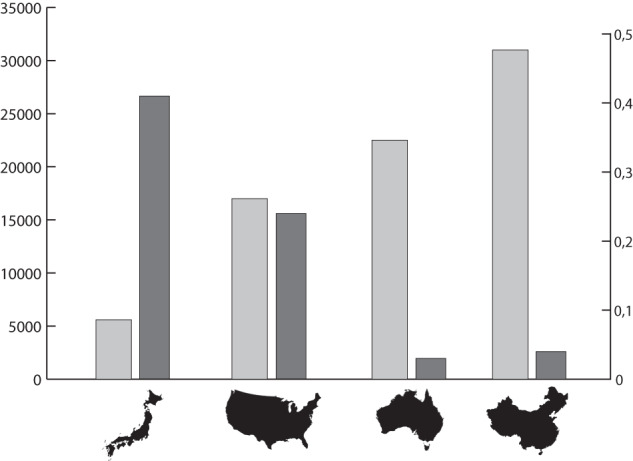
Possible influence of academic tradition in reporting plant hybrid zones. Percentage of hybrid zones (dark grey) from four countries with respect to the total number recorded in Abbott (2017) compared with the estimated number of vascular plants in each of them (light grey). From left to right, Japan, United States, Australia, China.

However, the puzzle is not necessarily that HZs are rarer in plants, but that they are less reported in the literature, which may be influenced by biases associated with different scientific disciplines or academic traditions. For example, in Abbott’s (2017) compilation, 16.8% (23) of the 137 HZs are from Japan, which contains c. 5600 species of vascular plants (0.41 of the Japanese flora reported to be involved in HZs) (https://www.cepf.net/our-work/biodiversity-hotspots/japan/species) (Fig. 2). These figures are comparable to those from the USA, from which 42 HZs are recorded (30.6% of Abbott’s compilation) from a total of c.17,000 vascular plants (0.24 of the US flora). But they contrast with 7 HZs recorded from Australia (5.1% of the compilation), whose flora includes c. 22,500 species (0.03 of the Australian flora) (https://www.dcceew.gov.au/science-research/abrs/publications/other/numbers-living-species/discussion-plants), and 13 HZs compiled from China (9.4% of 137), whose flora includes c. 31,000 species (0.04 of the Chinese flora) (http://flora.huh.harvard.edu/china/mss/plants.htm). These contrasting patterns may be influenced by different biomes, as HZs reported from tropical regions are scarce (Turchetto et al. 2022), although these four countries all include subtropical or tropical areas, or by different levels of knowledge. However, different academic traditions may also have influenced the focus on hybridisation studies, as HZs.

### Lack or deficit of spatial structure

Hybridisation events that result in sterile F1 hybrids probably contribute to our puzzle. Although they are common or even the norm in plants, they do not constitute HZs because they are occasional and sometimes ephemeral: “Occasional hybridisation between recognisable species or subspecies is, therefore, the rule in flowering plants” (Stebbins 1959). This is reflected in floras (Ellstrand et al. 1996; Whitney et al. 2010; Marques et al. 2018), especially those that have been thoroughly studied for hybridisation (Stace et al. 2015). Considering the plant species recorded in the British Isles, 69% of the possible hybrids for the genus *Epilobium* (31) have been reported there, 40% for *Euphrasia* (69 hybrids; Preston and Pearman 2015). In the former genus, hybrids are mostly sterile, whereas in the latter, hybrids are usually highly fertile (Stace et al. 2015). Yet, no HZ has been reported for either of these genera, illustrating that the puzzle persists in the most extensively studied region for hybridisation in plants.

Dispersal of progenitors is thought to play a critical role in the persistence of HZs (Barton and Hewitt 1985; Thomas et al. 2008; Brennan et al. 2009; McEntee et al. 2020; but see Curry 2015). Dispersal of hybrid embryos (seeds) across and out of the HZ has been less considered (Tochigi et al. 2021) and may also play a role in the puzzle. If hybrid plant embryos or juveniles tend to disperse more than animals, this would contribute to spatially less structured hybridisation scenarios, leading to a geographically diffuse distribution of hybrid offspring, consistent with Harrison’s (1993) description of plant HZs. This is conceivable when comparing plants to animal groups traditionally considered to have low vagility such as amphibians and insects, but it is unclear whether it could apply to avian HZs. We do not have direct comparative evidence to support the importance of the mobility of plant hybrid embryos, but there are several features in plants that may have favoured geographically diffuse hybridisation scenarios. Seed dispersal capacity varies with the nature of dispersal mechanisms and their adaptations (Van der Pijl 1982). However, the prevailing view in plants is that long-distance dispersal, even if infrequent, is important for range expansion over time (Sanmartín and Ronquist 2004; Hampe 2011), and it is not precluded by the lack of specific adaptations (Green et al. 2022). Another feature that could contribute to a less structured HZ, or even no HZ, associated to hybrid seed dispersal is the high frequency of hermaphrodite flowers compared to animals, which mostly have separate sexes. Because some degree of self-compatibility is common in plants, individual (hybrid) plants can be founders of a population beyond the contact zone of the parental species or populations. This is in contrast to most vertebrate groups, where there are separate sexes and offspring are highly dependent on their progenitors in the early stages of life.

The dispersal of hybrid embryos may also depend on the HZ model. Barton and Hewitt (1985) suggested that plants “where hybrid populations are apparently isolated from one or both parents”, might be an exception to the tension zone model. Although some plant HZs fit the tension model (Šmíd et al. 2020; Natola et al. 2022), cases where the HZ fits a mixed model or alternative models cannot be rejected are more common (Lexer et al. 2005; Brennan et al. 2009; Cruzan et al. 2021). Furthermore, in Abbott’s (2017) review on plant HZs, the mosaic zone was the most frequently reported or inferred. In the absence of intrinsic selection against all hybrid genotypes, some degree of environmental-dependent selection (e.g., Johnston et al. 2001; Rieseberg et al. 2003; Pinheiro et al. 2010; Jacquemyn et al. 2012), a substantial dispersal capacity, and rapid independence from progenitors would allow them to track efficiently their niches, which could lead to a dispersed distribution of hybrids. This profile could be enhanced by an abundant number of seeds produced per individual, which is common in plants.

A geographically diffuse distribution of hybrids between two parental species may not be the case at all sites where they come into contact. An example of this is between two bluebell species, *Hyacynthoides hispanica* and *H. non-scripta*. They form a hybrid zone in north-western Spain (Marquardt et al. 2022), but in Britain, where *A. hispanica* is introduced, a hybrid is produced that exhibits a geographically diffuse distribution (Ruhsam et al. 2020).

Preliminary results from landscape genetics in plants (reviewed in Sunderland et al. 2020) suggest that effective dispersal rates are lower in high quality habitats than in different types of human-modified landscapes. Because hybridisation is often associated with disturbed habitats (Anderson and Stebbins 1954; Grabenstein and Taylor 2018), this may facilitate more efficient dispersal of hybrid embryos. Human-mediated dispersal of hybrids may also play a role (Wichmann et al. 2009).

Polyploidy is a fundamental process in plants that often involves hybridisation (Soltis et al. 2009; Wendel 2015; Van de Peer et al. 2021). Some plant HZs include interploidy hybridisation (Šmíd et al. 2020; Arida et al. 2021), and despite the chromosomal barrier represented by different ploidies (Lafon‐Placette and Köhler 2016), the so-called triploid block can be partially overcome and interploidy hybrids can be formed that bridge progenitors (Husband 2004; Köhler et al. 2010). However, due to the strong postzygotic interploidy barriers (Comai 2005) and to the minority cytotype exclusion principle (Husband 2000), hybrids are usually not frequent at a given moment in time, implying that the interploidy contact zones are likely to fit at most diffuse HZs.

To advance this cause, future work in plant hybridisation should more explicitly address the spatial component and determinants of spatial structure, focusing on factors that influence extrinsic selection, such as mychorrizae (Jacquemyn et al. 2012).

### Lack or deficit of genetic structure

Hybrid swarms contain a variety of recombinant types, including early and later generation hybrids, and backcrosses (Harrison 1993; Abbott 2017). Hybrid swarms are often considered a type of hybrid zone when broadly defined (Harrison 1993), fitting unimodal HZs (Jiggins and Mallet 2000). However, if HZs are considered synonyms for clines (Barton and Hewitt 1985), hybrid swarms, which represent a poorly structured scenario, do not easily fit into HZs and could thus contribute to the puzzle.

Hybrid swarms are common in plants, which may be due in part to findings that postzygotic barriers, including failure to form hybrid seeds or sterility of hybrid offspring, are often less strong than prezygotic barriers in plants (Lowry et al. 2008; Baack et al. 2015) and that, unlike in most animals, prezygotic isolation does not evolve faster than postzygotic isolation (Widmer et al. 2009). In his seminal paper, Stebbins (1959) noted that some woody genera, such as *Quercus, Salix, Vaccinium, Arctostaphylos*, *Ceanothus, Eucalyptus*, and *Acacia*, are particularly prone to forming natural hybrid swarms, and that hybrid swarms could be intractable when apomixis and allopolyploidy are involved, e.g., in *Crataegus, Rubus, Potentilla, Taraxacum, Hieracium*. In Abbott’s (2017) review, “approximately 22% [of the case studies] were best described as hybrid swarms containing a wide range of hybrid types along with parental classes” (31 cases out of 137). Further studies of the fine genetic structure of plant HZs, especially unimodal zones, are needed to assess the importance of hybrid swarms in plants, including their evolutionary significance, e.g., whether explosive adaptive radiation is just an extraordinary consequence of hybrid swarms (Barrier et al. 1999; Meier et al. 2017) or a more frequent outcome, and whether it could be part of the speciation continuum (Seehausen 2013).

### Hybridisation between non-closely related species

The weaker postzygotic than prezygotic isolation barriers reported in plants suggest another possible avenue for exploring our question. In plants, it is conceivable that hybridisation between non-sister species, and in general between species that are not closely related, could be more common than in animals. However, how this could lead to fewer HZs in plants is less clear. One possibility is that whenever hybridisation occurs between more distantly related plant species, viable offspring may be scarce due to a higher probability of chromosomal, BDM, or cytonuclear incompatibilities and thus a lower probability of viable hybrid genotypes. If this were the case, viable hybrids should be scattered and the possibility of a classical hybrid zone would be diluted.

In a survey of phylogenetic proximity in the plant HZ literature selected by Abbott (2017), we considered four categories (1) non-sister species, (2) sister species (including subspecies of the same species), (3) species reported as closely related even if no explicit phylogeny was available, and those falling into the same polytomy, (4) insufficient or unavailable information. We found 56, 26, 33, and 22 cases fitting each of these categories, respectively (Table S3). Thus, non-sister species (40.88%) reached almost the same numbers as sister and closely related species together (43.07%), suggesting that hybrid zones between non closely related species are not uncommon in plants. To compare phylogenetic proximity in animal and plant HZs, we focused on vertebrates because they represent the most contrasting group in terms of HZs (Table 1). We examined the phylogenetic relatedness of hybridising vertebrate species involved in HZs in a subset of the HZs detected in the literature search in Table S2, i.e., those papers published between 2015 and 2022. A large percentage of the 101 HZs detected in this period involved hybridising species with close phylogenetic position (Table S4). Specifically, 65 (79.1%) involved sister or closely related species. This pattern was particularly strong for the 39 avian hybrid zones, of which 31 (79.4%) involved sister species and an additional 4 (10.2%) involved closely related species. Thus, our survey suggests that in vertebrates, hybridising species are often closely related, whereas this is not always the case in plants.

However, genetic divergence and phylogenetic relatedness between hybridising species have been widely explored as factors influencing viability of hybridisation and hybrid speciation, and the results are not entirely consistent. In animals, this relationship appears to be stronger (Coyne and Orr 1989; Mendelson 2003). For example, using 61 pairs of populations or species from different animal groups, Roux et al. (2016) found that the level of genetic divergence between hybridising species had a large effect on the probability that their hybrids would evolve reproductive isolation, and they further determined a specific range of molecular divergence values that allow or are associated with gene flow. In plants, intrinsic postzygotic isolation has also been found to be correlated with genetic divergence of hybridising species (Scopece et al. 2008; Widmer et al. 2009; Christie and Strauss 2018) and with phylogenetic proximity (Costa et al. 2013), but not in all groups (Moyle et al. 2004). Brown et al. (2023b) have recently reported, from a meta-analysis of the British angiosperm flora, that genetic distance between parental species is the strongest predictor of hybridisation. However, our point is not that hybridisation between non-closely related species is more common than between closely related species in plants, but that hybridisation between non-closely related species may be more common than in animals. These authors report that hybridisation between divergent species may still have important evolutionary consequences. Also, in groups with a propensity to hybridise, such as *Boechera*, hybridisation is largely unconstrained by phylogeny (Li et al. 2017), and in others, strong postzgotic barriers can be raised in recently diverged taxa (Sandstedt et al. 2021). Even in animals, specifically in *Drosophila*, Comeault and Matute (2018) found that populations of hybrids formed by parental species with intermediate levels of divergence were more likely to survive and exhibit effective premating reproductive barriers against each parent. Therefore, the potential influence of postzygotic instrinsic barriers on the lower number of reported plant HZs, while remaining a potential factor, should be investigated with more data. Comparative crossability tests of non-closely and closely related plant species known to be involved in HZs could allow assessment of this possible cause.

### Lability of plant hybrid zones over time

Plant HZs may be more labile —less stable in space and time— that animal HZs, in which case researchers would be less likely to detect the HZ at any given time.

Despite the relative stability over time often associated with HZs, dynamism is inherent to HZs in various ways (Wielstra 2019). Hybrid zones have been reported to be forced to move by factors such as environmental selection, competition, asymmetric hybridisation, dominance drive, hybrid fitness, human activity and climate change (Buggs 2007). The type of dynamism that could contribute to our puzzle is one that results in one of the two hybridising species being currently absent. If reconstructing past movements without direct observational evidence is challenging (Buggs 2007; Brown et al. 2023a), it is even more difficult to do in HZs that no longer exist as such, depending on, but not limited to, how old the origin of the HZ is (Gao et al. 2012). While there is no direct evidence that such HZ dynamics are more common in plants than in animals, there are numerous cases in plants where one or both hybridising species are no longer near hybrids. The Qinghai-Tibet Plateau homoploid hybrid species *Ostryopsis intermedia* (Wang et al. 2021) is currently hundreds of kilometres away from one of its progenitors (*O. davidiana*). Liu et al. (2014) proposed that its origin occurred during the Pleistocene, when a climate-driven southward range shift of *O. davidiana* brought it into contact with the other parental species. In the same vein, Kadereit (2015) argued that the ecogeographic displacement of hybrid lineages from parental lineages currently observed in some 28 revised plant studies may be due to climate-driven range shifts, particularly of parental lineages. Isolation favoured by such shifts could have driven homoploid hybrid speciation in dynamic climatic scenarios during the Pleistocene. The breakdown of a previous contact between hybridising species and hybrid offspring could also be caused by the extirpation of one or two of the parental populations due to demographic competition (Wolf et al. 2001). This is the most likely explanation why the eastern Spanish ‘orphan’ populations of the hybrid daffodil *N. ×perezlarae* are hundreds of kilometres east of its progenitor *N. cavanillesii* (Marques et al. 2010). Current continental disjunctions of hybrid and parental lineages represent extreme examples of the possible occurrence of ancient but no longer existing HZs, for example in allopolyploid cotton (*Gossypium*, Wendel 1989) and in peonies (*Paeonia*, Sang et al. 1995). The capacity of plant dispersal, which is relevant to the argument of plant HZs lability, is consistent with a comparative biogeographic study of plant and animal groups in the Southern Hemisphere (Sanmartín and Ronquist 2004). This study concluded that dispersal was more important than vicariance in explaining current distributions for plant groups, while the opposite was true for animal groups. This is also consistent with the notion of niche conservatism in plants (Donoghue 2008), which implies that plants efficiently track their niches through active dispersal. In contrast, in animal HZs, especially those that fit tension models, outcomes of HZ dynamics such as one progenitor being genetically swamped or demographically displaced by the other, or hybrid offspring dispersing and establishing outside the HZ, are unlikely.

In addition, HZs involving annual or short-lived plants may be particularly labile due to their fast life cycle and short generation times. In Abbott’s (2017) compilation of plant HZs, only 5.8% (8) of the cases involved annual species (Table S5). A possible explanation for this is that hybrid zones of short-lived organisms may be difficult to detect while they still contain parental and hybrid classes, especially if selection pressures have been strong. This is likely to be the case in the eastern Iberian populations of the annual weedy *Anacyclus*, where two species meet at different locations along the Mediterranean coast and hybridise, resulting in considerable phenotypic variability. However, while some of these populations are genetically similar to hybrid swarms, others are much more homogenous, presumably due to repeated rapid backcrossing in one direction (Agudo et al. 2023). Although attempts to characterise the spatiotemporal dynamics of HZs are common, comparative studies, especially including annual species, would help to assess whether plant HZs may indeed be less stable than animal HZs. These studies would also help us to assess whether, operationally, the HZ concept should be strictly time-bound, i.e., only apply to situations where both progenitors and hybrid offspring are currently occurring.

### Botanists’ perception of hybridisation

Harrison (1993) noted that “Presumably as a consequence of their different experiences with hybridisation in natural populations, botanists and zoologists have developed rather different views of the ‘evolutionary role’ of hybridisation”. There are many references in the literature to Mayr’s (1942) ideas about the evolutionary role of hybridisation, which he considered negligible (e.g., Sætre 2013; Harrison and Larson 2014). The influence of Mayr’s views was strong among evolutionary biologists working with animal groups, but not among those working with plants. Zoologists largely accommodated observations of natural hybridisation within a hybrid zone framework until the 21st century. In contrast, botanists over the last century have tended to view hybridisation and introgression as potential driving mechanisms for generating new diversity and promoting adaptation (Lotsy 1916; Anderson 1949; Stebbins 1959; Grant 1981), thus playing a creative role in evolution. These contrasting views of the two scientific communities likely influenced zoologists to find and report higher numbers of HZs. An anecdotal, or perhaps not, example of these different perceptions concerns paloverde trees in California, where botanists describe hybridisation between *Cercidium floridum* and *C. microphyllum* (Fabaceae) without mentioning the term hybrid zone in their paper (Jones et al. 1998), while entomologists explicitly refer to a paloverde hybrid zone in the title of their paper focusing on beetles (Fox et al. 1997).

However, biological differences may also have contributed to these contrasting views too. Abbott (2017) argues that “only early generation hybrids are likely to be recognised as such, with later generation backcrosses resembling one or both parents missed” and that “many bimodal HZs comprising mainly backcrosses and parental types will not be recognised as HZs in the wild based on morphological analysis alone”. Recognition of hybridisation in floristic or faunistic studies, i.e., studies that are not focused on the organism in question but on areas, may depend strongly on whether F1 hybrids are found. Unlike backcrosses or even late-generation hybrids, F1 hybrids may be distinguished morphologically by two types of patterns, depending on the character: intermediacy (Gottlieb 1972; Wilson 1992; Rieseberg 1997) and heterosis (Lippman and Zamir 2007; Chen 2013). Intermediacy is the main cue for recording hybrids in floras (Ellstrand et al. 1996; Whitney et al. 2010). It is therefore possible, as Abbott suggests, that while botanists frequently observe F1 hybrids in the wild, they may not observe HZs —even if they exist— if they consist of a majority of genotypes close to the hybridising species. A HZ with this type of genetic structure and a diffuse spatial structure, which are common in plants (see above), would be even more difficult to detect. Mimura and Suga (2020) have recently proposed another, quite opposite, cause for the predominance of individuals phenotypically similar to the parental species in a Japanese *Rubus* hybrid zone, suggesting that this may contribute to the rarity of reported plant HZs: selection pressures on leaf traits that causes steeper morphological than molecular clines.

In addition, the frequency of hybrids in plants may also play a role in the puzzle. Because botanists do not view hybrids as oddities, but rather as something to be expected, finding one hybrid plant individual does not necessarily indicate that other hybrids should be found or sought. Thus, botanists working with non-model organisms may have overlooked HZs, especially bimodal and spatially dispersed ones. To avoid neglecting existing HZs, botanists should consider them as a possible outcome of hybridisation when first detecting hybrids, rather than just recording them on an individual basis.

### Deficit of population genetic data

The failure to study plant hybridisation under HZ frameworks may also be due to insufficient genetic data for some plant groups. Abbott (2017) pointed out that “population genetic analysis is required to confirm the existence and structure of […] hybrid zones, and this is likely to have imposed a constraint on the number of hybrid zones detected, due to the scientific and financial resources required for such analysis”. This factor may also have influenced the number of reported plant HZs. However, to assess how this might affect plant and animal studies differently, additional factors might be considered. Number of species is one of these. Resources for studying non-model species may be a more limiting factor in a clade with about 390,000 species (vascular plants) than in one with about 60,000 (vertebrates). This is not the case for insects, where classical HZs have long been known (e.g., Hewitt 1975; Moran 1979).

Population genetic studies are important for the plant sciences community in general, and crucial for conservation (Ellstrand and Elam 1993). However, in (micro) evolutionary-oriented studies involving hybridisation, population genetics may have ‘competed’ over the past three decades with phylogeography, a discipline that was explicitly introduced to merge the fields of population genetics and phylogenetics (Avise et al. 1987). There are many phylogeographic studies dealing with plant hybridisation that do not adopt the hybrid zone framework (e.g., Gutiérrez Larena et al. 2002; Choler et al. 2004; Owens et al. 2016; Schneeweiss et al. 2017). However, a survey in Clarivate Web of Science using the subject search terms ‘hybrid zone’, ‘hybridisation’, ‘population genetics’ and ‘phylogeography’ in various combinations with or without ‘plants’, indicates that phylogeographic studies have not caused a reduction in the number of HZ studies using population genetics (Table S6). ‘Phylogeography’ is mentioned less often than ‘population genetics’ in papers mentioning either ‘hybrid zone’ or ‘hybridisation’, even more so for plants than for animals. Furthermore, in searches using either ‘population genetics’ or ‘phylogeography’, hits are reduced when ‘plants’ is added to the search terms. However, this reduction is stronger when ‘hybrid zone’ is included in the search instead of ‘hybridisation’, which is consistent with the alluded reports of hybridisation without mention of hybrid zones in plant studies discussed in the previous section. Therefore, these literature searches do not indicate that phylogeography plays a role in the puzzle. The important issue may be sampling depth. Population genetic studies require and usually include good sampling, whereas phylogeographic studies do not always include it for putative hybrid individuals. Identification and, if so, taxonomic description of hybrids does not usually involve dense sampling either.

The lack of sufficient data (including sampling) and appropriate analytical approaches could delay the identification of a HZ. Re-analysis of isozyme data from eastern Mediterranean *Senecio* populations unveiled a HZ lying across a c.170 km aridity gradient (Abbott et al. 2018), which had been missed in a previous study. Our own work on non-model plants provides another example of a late discovery of a HZ. A few samples from a peripheral population of a coastal sand dune plant species —*Armeria pungens*— were initially attributed to introgression from a congener in phylogeographic studies without fine spatial analysis (Piñeiro et al. 2007, 2011). Only when the area could be finely explored, phenotypic and genotypic diversity could be assessed (Nieto Feliner et al. 2019), and genomic cline analysis could be performed (Villa-Machío et al. 2023), was it possible to collect enough information to indicate the presence of a hybrid zone. To minimise the possible impact of poor data on the puzzle, future studies should ensure representative sampling and appropriate analytical approaches, including cline analysis, whenever hybrid plants are detected.

## Conclusions

There is no way to conclusively solve the puzzle except by re-examining both all reported plant HZs and cases of plant hybridisation where no HZ was reported, a task that is out of reach. However, a review of the literature for possible factors, based on both plant peculiarities and the ideas and practices of plant and animal scientists, may give clues to the big picture. Nevertheless, the proposed possible causal factors are non-mutually exclusive working hypotheses. With this approach, our conclusion is that the causes underlying the scarcity of recorded plant hybrid zones, despite the propensity of plants to hybridise, are most likely multifactorial. The potential factors listed above are likely to be complementary, partially overlapping and interacting. Factors such as the ‘botanical’ perception of hybridisation, which would be probably supported by a majority of botanists over decades, apply to most cases, and thus the weight of this factor in explaining the puzzle is high. Others may be more case-dependent. Looking at the features of plant hybrid zones collected or inferred by Abbott (2017)—and from the features we looked at additionally—it seems that there are no absolute regularities, either in general or in relation to the type of hybrid zone. However, considering the biological features that influence hybridisation, where plants have been reported to be more or less different from animals, the best-case scenario for departing from a classical HZ structure may involve weaker postzygotic than prezygotic barriers (Lowry et al. 2008; Widmer et al. 2009; Baack et al. 2015) and environmental-dependent selection. The latter was emphasised by Arnold (1997) and supported by several studies (Johnston et al. 2001; Rieseberg et al. 2003; Pinheiro et al. 2010; Jacquemyn et al. 2012). In scenarios that include these two features and a moderate seed dispersal capacity, even hybrid genotypes that are less fit than parental species in their close proximity and habitat would have improved chances of survival in non-close proximity and/or different habitats. Such scenarios could lead to dispersed spatial structures, close to the mosaic zone model that Abbott (2017) concludes is the most frequent in his review, to the ‘evolutionary novelty model’, or —depending on the frequency of successful hybrid genotypes— to no HZ at all, and would also fit Harrison’s (1993) notion of diffuse plant HZs.

Beyond this possible scenario, it is difficult to generalise further causes for the puzzle discussed here. With the exception of a few plant groups in which HZs have been studied in detail (e.g., *Helianthus, Iris, Mimulus, Picea, Populus, Quercus, Senecio*), the information provided in studies is insufficient to investigate the involvement of possible causes for the puzzle. Therefore, even though some plant features suggest that plant hybrid zones are less common than animal hybrid zones, we cannot at this point conclusively reject the null hypothesis that the low number of plant hybrid zones is an artefact, and we encourage future studies to address this issue in the light of forthcoming more comprehensive data.

### Supplementary information

Supplemental Table 1

Supplemental Table 2

Supplemental Table 3

Supplemental Table 4

Supplemental Table 5

Supplemental Table 6
