# Supplementary material for: The puzzle of plant hybridisation: a high propensity to hybridise but few hybrid zones reported
Source: Heredity (Edinb). 2023 Oct 27;131(5-6):307–15. doi: 10.1038/s41437-023-00654-1 (PMC10673867; doi:10.1038/s41437-023-00654-1)
Supplement: Supplementary file 1 — Supplemental Table 1 [file 41437_2023_654_MOESM1_ESM.pdf]

Table S1.— Hybrid zones involving vascular plant species detected in a literature survey conducted in Google Scholar of papers published in all journals from 1970 to 2022 that included ‘hybrid zone’ in their title (unpublished theses and preprint manuscripts not considered).

	Taxa	Group	Source for the hybrid zone	Other papers on the same HZ
1	Acer pictum subsp. mono x Acer truncatum	Angiosperms	https://doi.org/10.1038/s41598-022-17538-9	
2	Aconitum lasiocarpum x A. variegatum	Angiosperms	https://doi.org/10.2478/abcsb-2013-00015	
3	Aesculus pavia x A. sylvatica	Angiosperms	https://doi.org/10.1111/j.1558-5646.1990.tb05233.x	IJPS-08, AJB-06,
4	Alnus glutinosa s. str. x A. rohlenae	Angiosperms	https://doi.org/10.3390/genes11070770	
5	Alnus glutinosa x A. incana	Angiosperms	https://doi.org/10.2307/1941682	
6	Anthoxanthum alpinum (diploid and tetraploid populations)	Angiosperms	https://doi.org/10.1111/j.1469-8137.1996.tb01921.x	
7	Antirrhinum majus pseudomajus x A. m. striatum	Angiosperms	https://doi.org/10.1080/23818107.2018.1545142	JEvoBiol-11, ArthropInterct-08,
8	Aquilegia flavescens x A. formosa	Angiosperms	https://doi.org/10.1139/cjb-2020-0015	
9	Armeria pseudoarmeria x A. welwitschii	Angiosperms	https://doi.org/10.1007/s00606-008-0019-x	
10	Armeria pubigera x A. beirana	Angiosperms	https://doi.org/10.3989/ajbm.2007.v64.i2.180	
11	Artemisia tridentata subsp. tridentata and A. t. subsp. vaseyana	Angiosperms	https://doi.org/10.1111/j.1558-5646.1997.tb02391.x	AJB-91, AJB-95, AJB-95, BJLS-05, AJB-07, BiochSystEcol-99, EvoEcoRes-99, AJB-99, IJPS-98, Oecologia-01, CanJBot-98,
12	Asclepias exaltata x A. syriaca	Angiosperms	https://doi.org/10.3732/ajb.1100272	
13	Banksia robur x B. oblongifolia	Angiosperms	https://doi.org/10.1093/aob/mcq001	
14	Boechera stricta, two subspecies	Angiosperms	https://doi.org/10.1038/s41559-017-0119	
15	Borrichia frutescens x B. arborescens	Angiosperms	https://doi.org/10.3732/ajb.91.11.1757	
16	Bursera cuneata, B. palmeri and B. bipinnata	Angiosperms	https://doi.org/10.1371/journal.pone.0260382	

17	Castilleja miniata, C. rhexiifolia, C. sulphurea	Angiosperms	https://doi.org/10.3732/ajb.0800357	
18	Cattleya coccinea x C. brevipedunculata	Angiosperms	https://doi.org/10.1111/boj.12437	
19	Cerasus leveilleana x C. sargentii	Angiosperms	https://doi.org/10.1111/1442-1984.12311	
20	Clarkia nitens x C. speciosa polyantha	Angiosperms	https://doi.org/10.1002/j.1537-2197.1983.tb10848.x	
21	Dactylorhiza incarnata ssp. cruenta x D. lapponica	Angiosperms	https://doi.org/10.1038/sj.hdy.6800643	
22	Dendrocalamus pendulus x G. scortechinii	Angiosperms	Gardens' Bulletin Singapore 63(1 & 2): 375–383. 2011	
23	Dryas integrifolia x D. octopetala	Angiosperms	https://doi.org/10.1657/1523-0430(06-018)[HOYE]2.0.CO;2	
24	Epimedium diphyllum x E. sempervirens var. rugosum	Angiosperms	https://doi.org/10.5091/plecevo.2012.637	
25	Eriophyllum lanatum var. achilleoides x E. l. var. leucophyllum	Angiosperms	https://doi.org/10.3120/0024-9637-55.4.269	
26	Erythronium oregonum x revolutum	Angiosperms	https://www.jstor.org/stable/41424670	
27	Eucalyptus risdonii x E. amygdalina	Angiosperms	Australian J Bot 34: 305 - 329	
28	Fraxinus excelsior x F. angustifolia	Angiosperms	https://doi.org/10.1186/1471-2148-6-96	ME-06,
29	Helictotrichon parlatorei x H. setaceum subsp. petzense	Angiosperms	Schlechtendalia, 12, 69-83 (2004)	
30	Hippophae rhamnoides ssp sinensis x H. neurocarpa ssp neurocarpa	Angiosperms	J Northwest Normal Univ. (Natural Science), 44, 73-77 (2008)	
31	Hyacinthoides hispanica x H. non-scripta	Angiosperms	https://doi.org/10.1093/evolinnean/kzac003	
32	Ipomopsis aggregata x I. tenuituba	Angiosperms	https://doi.org/10.1002/ajb2.16067	AJB, AmNat_97, Evo-98, Oecologia-05, Evo-98, Evo-03, AJB-97, Oecologia-02, AJB-00,
33	Iris fulva, I. brevicaulis, I. hexagona	Angiosperms	https://doi.org/10.1111/j.1558-5646.1993.tb02165.x	Evo_94,
34	Iris hexagona x I. fulva	Angiosperms	https://doi.org/10.1111/mec.14481	Oikos-03,
35	Juniperus virginiana x J. horizontalis	Angiosperms	https://doi.org/10.1139/b83-301	
36	Leucosceptrum japonicum and L. stellipilum	Angiosperms	https://doi.org/10.1007/s00606-014-1134-5	

37	Lilium japonicum x L. auratum	Angiosperms	https://doi.org/10.2503/hortj.OKD-074	
38	Lilium meleagrinum, L. gongshanense, L. saluenense	Angiosperms	https://doi.org/10.3389/fpls.2020.576407	
39	Linum marginale ”bog” and ”hill” ecotypes	Angiosperms	https://doi.org/10.1007/s004420050937	
40	Metrosideros polymorpha var. incana x M.p. var. Glaberrima	Angiosperms	https://doi.org/10.1038/hdy.2016.40	
41	Nothofagus nervosa x N. obliqua	Angiosperms	https://doi.org/10.1007/s11295-017-1132-1	
42	Orchis anthropophora, O. militaris, O. purpurea	Angiosperms	https://doi.org/10.1111/j.1469-8137.2011.03913.x	
43	Orchis mascula x O. pauciflora	Angiosperms	https://doi.org/10.1023/A:1026534425741	
44	Origanum husnucan-baseri × O. saccatum	Angiosperms	https://doi.org/10.5735/085.057.0120	
45	Oxytropis diversifolia x O. leptophylla	Angiosperms	https://doi.org/10.1002/ece3.9351	
46	Penstemon newberryi x P. davidsonii	Angiosperms	https://doi.org/10.1111/j.0030-1299.2008.16573.x	
47	Phlox drummondii ssp. drummondii x P.d. ssp. mcallisteri	Angiosperms	https://doi.org/10.1002/j.1537-2197.1985.tb08398.x	
48	Phyllodoce caerulea x P. aleutica	Angiosperms	https://doi.org/10.1111/j.1420-9101.2007.01476.x	
49	Picea glauca × P. engelmannii	Gymnosperms	https://doi.org/10.1111/eva.12525	NewPhytol,
50	Picea sitchensis × P. glauca	Gymnosperms	https://doi.org/10.1111/mec.12007	NewPhytol, AMB,
51	Pinus californiarum x P. edulis	Gymnosperms	Oecologia 101: 29-36 (1995)	
52	Pinus contorta x P. banksiana	Gymnosperms	https://doi.org/10.1139/cjfr-2018-0428	JChemEcol, EvoApp-12,
53	Pinus massoniana x P. taiwanensis	Gymnosperms	J Nanjing Forest Univ, 36(6), 143-146 (2012)	
54	Pinus pumila x P. parviflora var.pentaphylla	Gymnosperms	https://doi.org/10.1007/BF02344555	ME-08,
55	Pinus sibirica and P. pumila	Gymnosperms	https://doi.org/10.1007/s10342-019-01254-7	AnForRes-07, AnForRes-08,
56	Pinus strobiformis x P. flexilis	Gymnosperms	https://doi.org/10.1111/eva.12795	CommBiol,
57	Piriqueta caroliniana (caroliniana x viridis morphotypes)	Angiosperms	https://doi.org/10.1111/j.1469-8137.2005.01410.x	IJPS-04,

58	Plathymenia reticulata ecotypes	Angiosperms	https://doi.org/10.1002/ece3.8540	
59	Polystichum munitum x P. imbricans	Pteridophytes	https://doi.org/10.2307/2656653	
60	Populus alba x P. tremula	Angiosperms	https://doi.org/10.1111/mec.13850	SciRep, ME, NewPhyt-07,
61	Populus angustifolia x P. fremontii	Angiosperms	https://doi.org/10.1111/j.1558-5646.1991.tb02641.x	
62	Populus balsamifera x P. deltoides	Angiosperms	https://doi.org/10.1002/ece3.1029	PLOSone, ECE,
63	Primula vulgaris × P. veris	Angiosperms	https://doi.org/10.1078/0367-2530-00149	ActaBiolSzeg-05,
64	Prosopis alba x P. nigra	Angiosperms	https://doi.org/10.2478/sg-2020-0007	
65	Prunella grandiflora x P. vulgaris	Angiosperms	https://doi.org/10.2307/2656999	
66	Quercus affinis x Q. laurina	Angiosperms	https://doi.org/10.1111/1442-1984.12109	
67	Quercus gambelii x Q. grisea	Angiosperms	https://doi.org/10.1111/j.1558-5646.1997.tb03658.x	Oecologia-96,
68	Quercus hypoleucoides, Q. scytophylla, Q. sideroxyla	Angiosperms	https://doi.org/10.1093/aob/mcp301	
69	Quercus liaotungensis x Q. mongolica	Angiosperms	https://doi.org/10.1093/jpe/rtv023	
70	Quercus wislizeni, Q. parvula, Q. agrifolia, Q. kelloggii	Angiosperms	https://doi.org/10.1111/j.0014-3820.2004.tb01643.x	
71	Ranunculus austro-oreganus x R. occidentalis	Angiosperms	https://doi.org/10.1111/evo.14381	
72	Ranunculus cantoniensis complex	Angiosperms	https://doi.org/10.1111/boj.12113	
73	Rhododendron japonoheptamerum var. hondoense x R. j. var. kyomaruense	Angiosperms	https://doi.org/10.1007/s11295-016-1084-x	
74	Rhododendron ponticum × R. caucasicum	Angiosperms	https://doi.org/10.1046/j.1365-294X.2003.01942.x	
75	Rorippa amphibia x R. sylvestris	Angiosperms	https://doi.org/10.1007/s00606-004-0131-5	
76	Salix purpurea x S. helvetica	Angiosperms	https://doi.org/10.1002/ece3.2470	
77	Salix sericea Marshall x S. eriocephala	Angiosperms	https://doi.org/10.1046/j.1365-294X.2000.00757.x	

78	Schiedea menziesii x S. salicaria	Angiosperms	https://doi.org/10.1371/journal.pone.0024845	
79	Senecio aethnensis x S. chrysanthemifolius	Angiosperms	https://doi.org/10.1111/mec.13618	Hered, Evo_05, NewPhyt-09, AJB-05, AnnBot-12,
80	Senecio vernalis x S. glaucus	Angiosperms	https://doi.org/10.1080/17550874.2018.1496366	
81	Sideritis serrata x S. bourgaeana	Angiosperms	https://doi.org/10.1093/oxfordjournals.aob.a088009	
82	Silene vulgaris x S. uniflora ssp. petraea	Angiosperms	https://doi.org/10.1139/b00-113	
83	Typha latifolia x T. angustifolia	Angiosperms	https://doi.org/10.1038/s41437-020-0307-y	
84	Viola rossii x V. bissetii	Angiosperms	https://doi.org/10.1111/1442-1984.12054	
85	Yucca baccata x Y. schidigera	Angiosperms	https://doi.org/10.1111/j.1558-5646.1998.tb02019.x	
86	Yucca brevifolia x Yucca jaegeriana	Angiosperms	https://doi.org/10.3389/fpls.2020.00640	AJB,
