# Supplementary material for: The puzzle of plant hybridisation: a high propensity to hybridise but few hybrid zones reported
Source: Heredity (Edinb). 2023 Oct 27;131(5-6):307–15. doi: 10.1038/s41437-023-00654-1 (PMC10673867; doi:10.1038/s41437-023-00654-1)
Supplement: Supplementary file 2 — Supplemental Table 2 [file 41437_2023_654_MOESM2_ESM.pdf]

Table S2.— Hybrid zones involving animal species detected in a literature survey conducted in Google Scholar of papers published in all journals from 1970 to 2022 that included 'hybrid zone' in their title, arranged in three groups: vertebrates, insects, other invertebrates (unpublished theses and preprint manuscripts not considered).

	Taxa	Group	Source for the hybrid zone	Other papers with same HZ
	**VERTEBRATES**			
1	Eptesicus fuscus (two mitochondrial lineages)	bats	https://doi.org/10.1644/06-MAMM-A-228R1.1	
2	Myotis lucifugus x M. yumanensis	bats	https://doi.org/10.1139/z83-268	
3	Rhinolophus affinis himalayanus x R. a. macrurus	bats	https://doi.org/10.1186/1471-2148-14-154	
4	Uroderma bilobatum davisi x U. B. convexum	bats	https://doi.org/10.2307/2407899	
5	Alectoris rufa x A. graeca	birds	https://doi.org/10.2307/4089367	
6	Ammodramus caudacutus x A. nelsoni	birds	https://doi.org/10.1186/s12862-016-0635-y	BMCEV, BJLS, Auk, ECE, ConsGen, Auk, WilsonJOrnith-11,
7	Anairetes reguloides x A. nigrocristatus	birds	https://doi.org/10.1111/mec.12836	
8	Aphelocoma woodhouseii subspecies	birds	https://doi.org/10.1093/auk/uky018	
9	Aquila clanga x A. pomarina	birds	https://doi.org/10.1186/1471-2148-11-100	
10	Baeolophus atricristatus x B. bicolor	birds	https://doi.org/10.1007/s00265-016-2126-y	AmMidlNat, Auk-90,
11	Branta hutchinsii x B. canadensis	birds	https://doi.org/10.1525/auk.2013.12196	
12	Callipepla californica x C. gambelii	birds	https://doi.org/10.1086/703158	ECE, Evo-07, BJLS-05,
13	Camarhynchus parvulus x C. pauper	birds	https://doi.org/10.1093/czoolo/61.1.181	
14	Catharacta maccormicki x C. antarctica lonnbergi	birds	J. Orn. 135 (1994): 48.	
15	Catharus ustulatus subspecies	birds	https://doi.org/10.1111/j.1558-5646.2007.00263.x	
16	Colaptes auratus auratus x C. a. cafer	birds	https://doi.org/10.1111/evo.14474	Auk-86, Evo-87, Auk-87, Auk-09, CanJZool-01, Condor-08, OrnApp-08,
17	Corvus (corone) corone) x (C. (c.) cornix	birds	https://doi.org/10.1111/evo.13970	NaEcEvol, Evo, JEvoBiol, Hered, JAvianBiol, Auk-92, ME-10, Ibis-05, ME-09, EvoEc-09, JAvianBiol-10, OrsScand-92, Jethol-08, BollZool-09, Ardea-94, BiocSystEcol-92, CanJZool-94, ItalJZool-98, Jornithol-98,
18	Cracticus tibicen (two plumage forms)	birds	https://doi.org/10.1525/auk.2011.11026	Emu-82,

19	Emberiza melanocephala x E. bruniceps	birds	https://doi.org/10.5253/arde.v105i1.a5	IranJBiol,
20	Ficedula albicollis and F. hypoleuca	birds	https://doi.org/10.1111/evo.12986	Evo, Jzool_99, Evo-09, Evo-12, Naturwissen-07,
21	Henicorhina leucophrys leucophrys x H. l. hilaris	birds	https://doi.org/10.1111/jeb.12876	
22	Hippolais polyglotta x H. icterina	birds	https://doi.org/10.1046/j.1095-8312.2003.00248.x	BehavProc-99, EvolBiol-11,
23	Icterus galbula x I. bullockii	birds	https://doi.org/10.1093/auk/ukaa044	RSocOpSci, Auk, Condor-96, JEvolBiol-06,
24	Jacana spinosa x J. jacana	birds	https://doi.org/10.1111/evo.13675	BMC Evol Biol,
25	Larus argentatus x L. cachinnans	birds	https://doi.org/10.1111/jeb.12404	
26	Larus occidentalis x L. glaucescens	birds	https://doi.org/10.1111/j.0014-3820.2000.tb00721.x	
27	Malurus melanocephalus subspecies	birds	https://doi.org/10.1111/evo.12457	
28	Manacus candei x M. vitellinus	birds	https://doi.org/10.1111/j.0014-3820.2001.tb01322.x	Science93, Evo-06, Evo-01, ME-09, ME-12,
29	Milvus milvus x M. migrans migrans	birds	https://doi.org/10.1371/journal.pone.0159202	
30	Motacilla alba alba x M.a. personata	birds	https://doi.org/10.1111/mec.14376	
31	Oporornis tolmiei x O. Philadelphia	birds	https://doi.org/10.1525/cond.2011.100182	
32	Paroaria baeri x P. gularis	birds	https://doi.org/10.1080/01584197.2016.1266447	
33	Passer domesticus x hispaniolensis	birds	https://doi.org/10.1002/ece3.5744	
34	Passer italiae x P. domesticus	birds	https://doi.org/10.1111/jeb.12652	JOrnith-96,
35	Passerina amoena x P. cyanea	birds	https://doi.org/10.1111/j.1365-294X.2010.04987.x	Evo-08,
36	Periparus ater melanolophus x P.a. aemodius	birds	https://doi.org/10.1002/ece3.8369	
37	Pheucticus melanocephalus x P. ludovicianus	birds	https://doi.org/10.1111/j.1365-294X.2009.04217.x	Willson Bull-74,
38	Phylloscopus collybita abietinus x P.c. tristis	birds	https://doi.org/10.1002/ece3.2782	
39	Phylloscopus collybita x P. brehmii	birds	https://doi.org/10.1046/j.1420-9101.2001.00273.x	
40	Pipilo maculatus x P. ocai	birds	https://doi.org/10.1002/ece3.375	
41	Platycercus adscitus x P. eximius	birds	https://doi.org/10.1038/s41437-018-0127-5	
42	Platycercus elegans elegans x P. e. flaveolus	birds	https://doi.org/10.1111/j.1600-048X.2013.00163.x	
43	Platycercus zonarius zonarius x P. z. semitorquatus	birds	https://doi.org/10.1525/auk.2008.07111	
44	Poecile atricapillus x P. carolinensis	birds	https://doi.org/10.1002/ece3.1774	Evo, Auk, ME, AmNat, ConsGenRes, CurrBiol, Evo, AnimBeh-03, Evo-07, BehEco-06, Auk-05, Auk-07,
45	Poephila acuticauda subspecies	birds	https://doi.org/10.1111/evo.14243	ME,
46	Pogoniulus bilineatus bilineatus x P. b. fischeri	birds	https://doi.org/10.1111/mec.15691	
47	Pogoniulus pusillus x P. chrysoconus	birds	https://doi.org/10.1111/mec.15691	
48	Puffinus mauretanicus x P. yelkouan	birds	https://doi.org/10.1038/s41598-019-51188-8	
49	Pyrrhura lepida coerulescens x P. l. anerythra	birds	https://doi.org/10.1007/s10336-015-1216-3	

50	Ramphocelus 'forms': flammigerus x icteronotus	birds	https://doi.org/10.1186/s12862-017-1096-7	
51	Rhegmatorhina hoffmannsi x R. berlepschi	birds	https://doi.org/10.1111/1evo.14437	
52	Selasphorus sasin × S. rufus	birds	https://doi.org/10.1093/auk/ukz049	
53	Setophaga coronata coronata x S. c. auduboni	birds	https://doi.org/10.1002/ece3.4469	ECE, Ornithology, PeerJ, JEvolBiol,
54	Setophaga townsendi x S. occidentalis	birds	https://doi.org/10.1111/jeb.13524	ECE, Evo-01, BehEco_00, PLOSOne-10, ProcB-08, BJLS-00,
55	Setophaga townsendi x S. virens	birds	https://doi.org/10.1111/jeb.12989	
56	Sphyrapicus nuchalis x S. varius	birds	https://doi.org/10.1111/jav.02717	
57	Sphyrapicus ruber x S. nuchalis	birds	https://doi.org/10.1111/mec.15043	ECE, Auk,
58	Sphyrapicus ruber x S. varius	birds	https://doi.org/10.1111/j.1600-048X.2012.05516.x	
59	Sphyrapicus ruber, S. nuchalis, and S. varius	birds	https://doi.org/10.1111/mec.16650	
60	Streptopelia vinacea x S. capicola	birds	https://doi.org/10.1093/beheco/arm018	PhilTransB-08,
61	Strix occidentalis caurina x S. o. occidentalis	birds	https://doi.org/10.1002/ece3.3260	ME-05, Condor-11,
62	Sula nebouxii x S. variegata	birds	https://doi.org/10.1111/j.1600-048X.2012.05660.x	
63	Thraupis episcopus x T. sayaca	birds	https://doi.org/10.1371/journal.pone.0270892	
64	Tockus erythrorhynchus rufirostris x T. e. damarensis	birds	https://doi.org/10.1093/auk/121.2.565	
65	Trochilus polytmus x T. scitulus	birds	https://doi.org/10.2988/0006-324X-128.1.111	
66	Vermivora chrysoptera x V. pinus	birds	https://doi.org/10.1007/s00265-007-0413-3	
67	Zosterops pallidus x Z. virens capensis	birds	https://doi.org/10.1093/biolinnean/blx012	
68	Bos taurus × Bos indicus	bovids	https://doi.org/10.1111/mec.12816	
69	Canis lycaon x C. latrans	canids	https://doi.org/10.1111/ddi.12543	
70	Canis rufus x C. latrans	canids	https://doi.org/10.1111/eva.12388	
71	Canis spp. X C. latrans	canids	https://doi.org/10.1002/ecs2.3320	Ecology, Oecologia, BiolCons, ME-12, CanFieldNat-03,
72	Mercenaria mercenaria x M. campechiensis	clams	https://doi.org/10.1111/j.1558-5646.1995.tb02240.x	MarBiol-89, MarBiol-93, MarBiol-96,
73	Crocodylus moreletii x C. acutus	crocodiles	https://doi.org/10.1111/mec.13694	
74	Cervus elaphus x C. nippon nippon	deer	https://doi.org/10.1111/j.1365-294X.1994.tb00086.x	Genetics99,
75	Odocoileus virginianus x O. hemionus)	deer	Biochemical Genetics, Vol. 30, Nos. 1/2, 1992	
76	Leopardus geoffroyi x L. tigrinus	felids	https://doi.org/10.1111/j.1365-294X.2008.03919.x	
77	Leopardus guttulus x L. geoffroyi	felids	https://doi.org/10.1111/jeb.13761	PLOSone
78	Lynx canadensis x L. rufus	felids	https://doi.org/10.1016/j.biocon.2014.07.016	
79	Abudefduf abdominalis x A. vaigiensis	fishes	https://doi.org/10.3390/d12020083	
80	Amphiprion chrysopterus x A. sandaracinos	fishes	https://doi.org/10.1002/ece3.6769	MolBiolRep-20,

81	Austrolebias charrua x A. reicherti	fishes	https://doi.org/10.1007/s10750-019-04104-0	Genes
82	Barbus barbus x B. meridionalis	fishes	https://doi.org/10.1111/j.1095-8312.1999.tb01929.x	JFishBiol-05, FreshwatBio-01,
83	Chondrostoma toxostoma toxostoma x C. nasus nasus	fishes	https://doi.org/10.1111/j.1095-8312.2005.00478.x	FrontZool-09, PLOSne-09,
84	Cottiusculus schmidti x C. nihonkaiensis	fishes	https://doi.org/10.1007/s12526-017-0771-7	
85	Cottus b. baird x C. caeruleomentum	fishes	https://doi.org/10.1093/jhered/92.4.309	
86	Cottus gobio x C. poecilopus	fishes	https://doi.org/10.1111/jai.12263	
87	Cyanoliseus patagonus subspecies	fishes	https://doi.org/10.1186/1742-9994-8-16	
88	Cyprinodon atrorus x Cyprinodon bifasciatus	fishes	https://doi.org/10.1111/j.1365-294X.2011.05433.x	Copeia-08,
89	Etheostoma bison x E. caeruleum	fishes	https://doi.org/10.1007/s10709-013-9707-8	
90	Fundulus heteroclitus ssp. heteroclitus x F. h. macrolepidotus	fishes	https://doi.org/10.1098/rsos.150285	ECE, JEvolBiol,
91	Fundulus heteroclitus x F. grandis	fishes	https://doi.org/10.1007/s11692-021-09553-x	
92	Fundulus majalis x F. similis	fishes	https://doi.org/10.1038/hdy.1995.18	
93	Gadus morhua (divergent populations)	fishes	https://doi.org/10.1046/j.1365-294X.2003.01819.x	CanJFisAqSci-05,
94	Gambusia holbrooki x G. affinis	fishes	https://doi.org/10.1002/ece3.2562	Evo-93,
95	Gymnogobius breunigii x G. castaneus	fishes	https://doi.org/10.1093/biolinnean/blab033	
96	Macquaria novemaculeata x M. colonorum	fishes	https://doi.org/10.1111/j.1095-8649.2011.03105.x	
97	Nematocharax venustus (two mitochondrial lineages)	fishes	https://doi.org/10.1111/jeb.13689	
98	Oncorhynchus clarki lewisi x O. mykiss	fishes	https://doi.org/10.1111/j.1365-294X.2004.02355.x	BJLS-12,
99	Rutilus rutilus x Abramis brama	fishes	https://doi.org/10.1111/j.1365-2427.2011.02604.x	
100	Salmo trutta (two lineages)	fishes	https://doi.org/10.1111/eva.13307	
101	Scophthalmus maximus	fishes	https://doi.org/10.1046/j.1365-294X.2004.02097.x	
102	Solea aegyptiaca x S. senegalensis	fishes	https://doi.org/10.1111/j.1365-294X.2011.05034.x	
103	Xiphophorus birchmanni x X. malinche	fishes	https://doi.org/10.1086/677033	JEvolBio-12, Copeia-03,
104	Vulpes vulpes patwin x introduced V.vulpes population	foxes	https://doi.org/10.1111/j.1365-294X.2010.04943.x	
105	Crinia laevis x C. victoriana	frogs	https://doi.org/10.1071/ZO9730277	
106	Discoglossus galganoi galganoi x D. g. jeanneae	frogs	https://doi.org/10.1038/s41437-020-0294-z	
107	Geocrinia laevis x G. victoriana	frogs	https://doi.org/10.2307/1446387	Hered-79, HerpConBiol-12, AmphRept-94, IsrJEcolEvol-96,
108	Geocrinia rosea populations	frogs	https://doi.org/10.1071/ZO08020	
109	Hyla arborea x H. orientalis	frogs	https://doi.org/10.1002/ece3.8527	

110	Hyperolius thomensis x H. molleri	frogs	https://doi.org/10.1093/biolinnean/blz131	
111	Litoria ewingii x L. paraewingi	frogs	https://doi.org/10.1111/evo.12140	ME, AustrJZool-72, AustrJZool-76, AustrJZool-83,
112	Odontophrynus cordobae x O. americanus	frogs	https://doi.org/10.1016/j.jcz.2021.07.001	
113	Pelodytes punctatus x P. hespericus	frogs	https://doi.org/10.1038/s41437-020-0294-z	
114	Pseudacris crucifer (two intraspecific mitochondrial lineages)	frogs	https://doi.org/10.1111/jeb.13017	
115	Pseudacris nigrita x P. fouquettei	frogs	https://doi.org/10.1002/ece3.2232	Copeia-80,
116	Pseudophryne bibroni x P. semiomarmorata	frogs	https://doi.org/10.2307/2407726	
117	Rana berlandieri x R.utricularia	frogs	https://doi.org/10.1111/j.1558-5646.1986.tb05714.xopen in new	
118	Ranidella insignifera x R. pseudinsignifera	frogs	https://doi.org/10.1038/hdy.1978.2	Hered-79
119	Ranitomeya imitator morphs	frogs	https://doi.org/10.1098/rspb.2014.1950	
120	Rhinella atacamensis x R. arunco	frogs	Rev.Chil. Hist. Nat. 86: 115-125 (2013)	
121	Sphaerodactylus nicholsi x S. townsendi	geckos	https://doi.org/10.1093/jhered/esz015	
122	Geomys bursarius × G. lutescens	gophers	https://doi.org/10.1111/j.1095-8312.1985.tb00397.x	
123	Thomomys bottae connectens x T. b. opulentus	gophers	https://doi.org/10.1111/j.1558-5646.1998.tb05164.x	
124	Thomomys bottae x Thomomys townsendii	gophers	https://doi.org/10.1111/j.1365-294X.1993.tb00093.x	
125	Erinaceaus europaeus x E. roumanicus	hedgehog	https://doi.org/10.1038/s41437-022-00567-5	ECE,
126	Eulemur fulvus rufus x E. albocollaris	lemurs	https://doi.org/10.1002/ajp.10033	
127	Eulemur rufifrons x E. cinereiceps	lemurs	https://doi.org/10.1007/s10764-015-9872-y	JEvolBiol, AmJPhyAnthrop-11,
128	Varecia variegata x V. rubra	lemurs	https://doi.org/10.1206/0003-0082(2002)376<0001:DRLFAH>2.0.CO;2	
129	Cnemidophorus tigris punctilinealis x C. t. marmoratus	lizards	https://doi.org/10.2307/1447696	Copeia-91,
130	Ctenophorus decresii x C. modestus	lizards	https://doi.org/10.1111/jeb.13772	
131	Lacerta lepida nevadensis x L. lepida lepida	lizards	https://doi.org/10.1111/jzs.12005	
132	Lacerta schreiberi (two divergent mtDNA lineages)	lizards	https://doi.org/10.1038/sj.hdy.6800823	PLoSOne-09,
133	Podarcis bocagei and Podarcis carbonelli	lizards	https://doi.org/10.1111/j.1439-0469.2009.00532.x	
134	Podarcis muralis (two lineages)	lizards	https://doi.org/10.1111/evo.14001	
135	Sceloporus cowlesi x S. tristichus	lizards	https://doi.org/10.1371/journal.pone.0025827	ME-17,
136	Sceloporus grammicus karyotypes	lizards	https://doi.org/10.1111/j.1558-5646.1995.tb05955.x	ME-08, Evo-95, ME-96, Herpetologica-98,
137	Sceloporus undulatus x S. woodi	lizards	https://doi.org/10.1093/jhered/est093	Evo-73,
138	Spalax sp cytotypes	moles	https://doi.org/10.1134/S1022795410100017	

139	Mus musculus musculus x Mus musculus domesticus	mouses	https://doi.org/10.1007/s00427-016-0550-7	ME, JHer, Jvirol, MERes, Hered, CurrZool, ECE, JGenEngeneerBiotech, Elife, ME, ProcRoySocB, JParasit, GenomeRes, Nature-86, Evo07, BMCEcEv08, JEvoBiol-93, Evo_92, Evo-04, JEvBiol_91, JEvBiol-04, Hered-08, ProcRB_94, ME-12, Evo-11, BJLS-05, Genetics-86, Parasit-93, Zeitsf Sauge-98, ME-11, Evo-04, Hered-01, ActaTher-03, GenRes-01, Evo-93, Evo-10, BJLS-05, Genetica-02, JZool-96, CytogGenRes-92, ProcB-91, VetParas-11, BJLS-05, Hered-93, BJLS-90, Genetics-02, BJLS-10, Genetics-96, Evo-80, CytogGenRes-02, Hered-99, JEvoBiol-08, Evo-98, BJLS-03, CytogenGenRes-11, JZooSysEvoRes-94, Genetica-97, ItalJZool-99,
140	Peromyscus californicus (two subspecies)	mouses	https://doi.org/10.1007/BF00173198	
141	Peromyscus leucopus (chromosomal races)	mouses	https://doi.org/10.2307/1382421	JHered-91,
142	Bathymodiolus antarcticus x B. thermophilus	mussels	https://doi.org/10.1186/1471-2148-13-21	
143	Bathymodiolus azoricus x B. puteoserpentis	mussels	https://doi.org/10.1046/j.0962-1083.2001.01401.x	ME-03, ISME-21
144	Mytilus edulis x chilensis	mussels	https://doi.org/10.1111/bij.12687	AqCons,
145	Mytilus edulis x M. galloprovincialis	mussels	https://doi.org/10.1007/s00227-019-3604-3	Genetics, JEvolBiol, MarineBiol, BJLS-08, Evo-03, MarBiol-05, Proteomics-07, MarBiol-03, MarBiol-00, JMoluscStu-09,
146	Mytilus edulis x M. trossulus	mussels	https://doi.org/10.1007/s00227-017-3249-z	PLOSone, MBE-02, MarBiol-04, Hered-09, ME-03, BiolBull-10,
147	Mytilus galloprovincialis (two lineages)	mussels	https://doi.org/10.1038/s41437-018-0174-y	
148	Mytilus trossulus x M. galloprovincialis	mussels	https://doi.org/10.1111/mec.13340	RussJGen, MarBiol-97, MarBiol-06, MarBiol-08,
149	Cynops pyrrhogaster (two lineages)	newts	https://doi.org/10.1093/jhered/esx085	JZoolSysEvRes,
150	Lissotriton boscai (two lineages)	newts	https://doi.org/10.1111/jeb.13562	
151	Triturus marmoratus x T. pygmaeus	newts	https://doi.org/10.1111/jzs.12439	ContrZool, ME-12,
152	Triturus vulgaris × T. montandoni	newts	https://doi.org/10.1046/j.1365-294X.2003.01880.x	
153	Alouatta palliata × A. pigra	primates	https://doi.org/10.1111/mec.14966	Jher, IntJPrimatol, AmJPhysPrimat,
154	Cercopithecus ascanius schmidti x C. mitis doggetti	primates	Am. J. Phys. Anthrop. (2000), pp. 138-138	
155	Hylobates lar x H. pileatus	primates	https://doi.org/10.1371/journal.pone.0264519	
156	Macaca mulatta x M. fascicularis	primates	https://doi.org/10.1111/iji.12405	ME-10,

157 Macaca tonkeana x M. hecki	primates	https://doi.org/10.1002/ajpa.10060	AJPrimat-97,
158 Papio cynocephalus x P. anubis	primates	https://doi.org/10.1098/rspb.2019.0431	IntJPrimatol, JAnimBeh, ME11,
159 Papio hamadryas anubis x P. h. hamadryas	primates	https://doi.org/10.1002/(SICI)1096-8644(199905)109:1<1::AI	PhysBiochZoo-12, AmNat-75,
160 Papio kindae x P. ursinus griseipes	primates	https://doi.org/10.1111/mec.15858	
161 Plecturocebus baptista x P. hoffmannsi	primates	https://doi.org/10.1590/1809-4392201803411	
162 Sapajus robustus x S. nigritus	primates	https://doi.org/10.1002/ajp.22696	
163 Oryctolagus cuniculus cuniculus x O. c. algirus	rabbits	https://doi.org/10.1111/mec.14494	ME, PeerJ, Genetics-06,
164 Ctenomys minutus chromosomal races	rodents	https://doi.org/10.1644/1545-1542(2002)083<0843:COAHZB	Jmammal-04,
165 Ambystoma californiense x A. tigrinum mavortium	salamanders	https://doi.org/10.1111/j.0014-3820.2004.tb01707.x	
166 Ambystoma maculatum (two lineages)	salamanders	https://doi.org/10.1093/jhered/esv042	
167 Ambystoma tigrinum nebulosum and A. t. mavortium	salamanders	https://doi.org/10.1046/j.1420-9101.1992.5030375.x	
168 Chioglossa lusitanica (two subspecies)	salamanders	https://doi.org/10.1111/jeb.13982	ME-04,
169 Ensatina eschscholtzii xanthoptica x E. e. platensis	salamanders	https://doi.org/10.1111/j.0014-3820.2005.tb01783.x	BMCEvoBiol-11,
170 Hydromantes ambrosii x H. italicus	salamanders	https://doi.org/10.1111/jbi.13621	
171 Lyciasalamandra antalyana x L. billae	salamanders	https://doi.org/10.1111/j.1420-9101.2005.01064.x	
172 Plethodon jordani, P. metcalfi, P. teyahalee	salamanders	https://doi.org/10.1111/j.1365-294X.2010.04796.x	
173 Plethodon teyahalee x P. shermani	salamanders	https://doi.org/10.1111/j.1365-2486.2009.01867.x	
174 Speleomantes italicus x S. ambrosii	salamanders	Ann Mus. Civ. Stor. Nat. Giac. Doria Genova, 97, 135-144 (2005)	
175 Triturus anatolicus x T. ivanbureschi	salamanders	https://doi.org/10.1002/evl3.9	
176 Triturus cristatus x marmoratus	salamanders	https://doi.org/10.1002/ece3.2676	Evo_91,
177 Triturus cristatus x T. carnifex	salamanders	https://doi.org/10.1111/csp2.12752	ECE, German J Herp, ConGenRes, Acta Herp,
178 Blarina brevicauda x B. hylophaga	shrews	https://doi.org/10.2307/1383214	Jmammal-99,
179 Sorex araneus (chromosome races)	shrews	https://doi.org/10.1111/j.1558-5646.1992.tb00624.x	RussJGen, ActaTherio, Evo-92, CompCytogen-17, ME-99, Hereditas-96, Evo-91, JEvoBiol-11, Hered-99, Jtheriol-07, ME-04, MamBiol-06, Hered-96, Hereditas-90, Hereditas-84, BJLS-06, Acta Theriol-03, CytogenGenRes-07, ComparCytogen-10, RussJGen-07, RussJGen-12, RussJGen-08, RussJGen 10, ActaTher-11, Genetika-08,
180 Sorex araneus x S. antinorii	shrews	https://doi.org/10.1038/hdy.2009.19	Hereditas-96, JEvBio-08,
181 Crotalus scutulatus x C. viridis	snakes	https://doi.org/10.3390/toxins8060188	

182	Crotalus viridis x C. oreganus concolor	snakes	https://doi.org/10.1111/evo.14612	
183	Natrix astreptophora x N. helvetica	snakes	https://doi.org/10.1093/biolinnean/blaa152	
184	Vipera aspis x V. latastei	snakes	Amphibia-Reptilia 31 (2010): 195-212	
185	Tamias ruficaudus ruficaudus x T. r. simulans	squirrels	https://doi.org/10.1111/j.1365-294X.2009.04196.x	
186	Tamiasciurus douglasii x T. hudsonicus,	squirrels	https://doi.org/10.1111/j.1365-294X.2011.05184.x	
187	Glaucomys sabrinu x G. volans	squirrels	https://doi.org/10.1139/cjz-2021-0086	
188	Gasterosteus aculeatus (two populations)	sticklebacks	https://doi.org/10.1111/evo.12917	JEvolBiol06,
189	Alytes obstetricans almogavarii x A. o. pertinax	toads	https://brill.com/view/journals/amre/41/1/article-p105_9.xml	
190	Anaxyrus americanus x A. hemiophrys	toads	https://doi.org/10.1371/journal.pone.0052819	Herpetologia-83,
191	Bombina bombina x B. variegata	toads	https://doi.org/10.1111/j.1558-5646.1998.tb05156.x	Evo_95, Evo-86, Evo-91, Evo-98, ProcB-97, BJLS-07, Experientia-85, JZooSysEvoRes-84, Evo-92, Ecology-02, ProcB-95, IsrJEcolEvol-96, Hered-05, North-WJZool-22,
192	Bufo americanus x B. fowleri	toads	https://doi.org/10.1643/0045-8511(2003)003[0034:VAAIIA]2.0.CO;2	
193	Bufo bufo x B. spinosus	toads	https://doi.org/10.1111/mec.14273	ME, Contrib. Zool., Amphibia-Reptilia,
194	Bufo siculus x B. balearicus	toads	https://doi.org/10.1186/1471-2148-10-232	
195	Lissotriton montandoni x L. vulgaris	triton	Herpetol. Rom. 5, 2011, pp.51-59	
196	Sternotherus depressus x S. peltifer	turtles	https://doi.org/10.1111/mec.14983	
197	Microtus arvalis x M. obscurus	voles	https://doi.org/10.1007/s00436-021-07134-7	RussJGen
198	Petrogale lateralis centralis x P. l. kimberleyensis	wallabies	https://doi.org/10.1071/ZO20052	
199	Neotoma bryanti x N. lepida	woodrats	https://doi.org/10.1002/ece3.7399	Evo, BMCEcolEvo,
200	Neotoma floridana and N. micropus	woodrats	https://doi.org/10.1093/jmammal/gyaa164	JHered,
201	Neotoma fuscipes x N. macrotis	woodrats	https://doi.org/10.1016/j.cub.2017.11.029	

INSECTS

1	Formica selysi x F. cinerea	ants	https://doi.org/10.1111/mec.13799	
2	Pogonomyrmex barbatus x rugosus	ants	https://doi.org/10.1111/evo.14481	
3	Tetramorium immigrans x T. caespitum	ants	https://doi.org/10.1111/1744-7917.12915	
4	Solenopsis geminata x S. xyloni	ants	https://doi.org/10.1111/j.0014-3820.2003.tb00364.x	
5	Solenopsis invicta x S. richteri	ants	https://doi.org/10.1111/j.1558-5646.1996.tb03583.x	ME-24, AnnEntSocAm-89, JEntSci-10,
6	Orconectes rusticus x O. propinquus	arthropods	https://doi.org/10.1111/j.0014-3820.2001.tb00635.x	
7	Apis mellifera capensis x A. m. scutellata	bees	https://doi.org/10.1038/sj.hdy.6801058	

8	Carabus lewisianus x C. albrechti	beetles	https://doi.org/10.1111/j.1095-8312.2005.00527.x	
9	Carabus maiyasanus x C. iwawakianus	beetles	https://doi.org/10.1002/ece3.1814	
10	Chauliognathus pensylvanicus	beetles	https://doi.org/10.2307/3566040	
11	Chyrsochus cobaltinus x C. auratus	beetles	https://doi.org/10.1111/j.0014-3820.2005.tb00976.x	AnEntSocAmer-01, Genetica-11,
12	Cicindela denverensis, Cicindela limbalis, Cicindela splendida	beetles	https://doi.org/10.1155/2012/398180	
13	Gonioctena aegrota x G. pseudogobanzi	beetles	https://doi.org/10.1111/j.1095-8312.2008.00972.x	
14	Gonioctena quinquepunctata and G. intermedia	beetles	https://doi.org/10.1111/jeb.13538	
15	Ips pini populations	beetles	https://doi.org/10.1007/s00049-007-0386-8	
16	Psacothea hilaris subsp. hilaris morphological types	beetles	https://doi.org/10.1093/ee/28.4.690	
17	Anartia fatima x A. amathea	butterflies	https://doi.org/10.1111/j.0014-3820.2002.tb01385.x	
18	Coenonympha macromma and C. gardetta	butterflies	https://doi.org/10.1111/eva.12925	
19	Danaus chrysippus chrysippus x D. c. dorippus	butterflies	https://doi.org/10.1111/j.1095-8312.1998.tb00349.x	ZJLS-97, Hered-03, BJLS-14,
20	Heliconius cydno cydnides x H. c. weymeri	butterflies	https://doi.org/10.1111/j.1365-294X.2012.05746.x	Biotropica-05,
21	Heliconius erato (Postman x Radiate metapopulations)	butterflies	https://doi.org/10.1126/sciadv.abb8617	Evo_89,
22	Heliconius erato chestertonii and H. e. venus	butterflies	https://doi.org/10.1111/j.1420-9101.2010.02001.x	ME-08,
23	Heliconius erato erato x H. e. hydara	butterflies	https://doi.org/10.1111/een.12386	JEvBiol-07,
24	Heliconius erato hydara and H. e. demophoon	butterflies	https://doi.org/10.1111/jeb.13499	
25	Heliconius erato hydara x H. e. petiverana	butterflies	https://doi.org/10.1111/j.0014-3820.2002.tb00125.x	
26	Heliconius erato x H. himera	butterflies	https://doi.org/10.1038/hdy.1997.189	
27	Hyalophora euryalus x H. columbia gloveri	butterflies	https://doi.org/10.2992/007.082.0204	
28	Leptidea sinapis x L. reali	butterflies	Eur. J. Entomol. 104: 667–674, 2007	
29	Limenitis arthemis astyanax x L. a. arthemis	butterflies	https://doi.org/10.1111/j.1558-5646.2008.00366.x	
30	Lycaeides melissa x L. idas	butterflies	https://doi.org/10.1111/mec.16469	ME-10,
31	Papilio glaucus x P. canadensis	butterflies	https://doi.org/10.1073/pnas.1714950115	ME, Jhered-89, InsSci-10, Oecologia-91, AmMidlNat-88, Ecography-02, GreatLakesEntom-03,
32	Papilio syfanius x P. maackii	butterflies	https://doi.org/10.7554/eLife.78135	
33	Pieris napi x P. bryoniae	butterflies	https://doi.org/10.1046/j.1365-2311.1997.00054.x	
34	Pontia daplidice x P. edusa	butterflies	https://doi.org/10.1111/j.1558-5646.1997.tb01479.x	
35	Pupillo glaucus glaucus x P. g. canadensis	butterflies	Am. Mid. Nut. 119: 366-379 (1988)	
36	Tatochila mercedis x T. sterodice	butterflies	https://doi.org/10.1093/aesa/83.2.107	AnnEntSocAm-90,

37	Allonemobius fasciatus x A. socius	crickets	https://doi.org/10.1046/j.1365-294x.2001.01215.x	Evo-98, Jhered-86, BJLS-08,
38	Gryllus firmus x G. pennsylvanicus	crickets	https://doi.org/10.1038/hdy.1986.55	Evo, ECE, ME, Evo97, Evo-89, Evo-02, Evo-82, Evo-09, BJLS-09, Hered-97, ME-08, Oikos-06, G3-15,
39	Ischnura elegans x I. graellsii	damselflies	https://doi.org/10.1002/ece3.4024	
40	Anopheles gambiae x A. coluzzii,	diptera	https://doi.org/10.1111/mec.13840	Malaria J,
41	Culex pipiens pipiens x C. p. quinquefasciatus	diptera	https://doi.org/10.1093/jmedent/45.2.276	JMedEnt-97, AmJTropMedHyg-08, JMedEnt-06,
42	Drosophila antonietae x D. serido	diptera	https://doi.org/10.1111/jeb.13934	
43	Drosophila mauritiana x D. simulans	diptera	https://doi.org/10.1111/jeb.12057	
44	Drosophila teissieri x D. yakuba	diptera	https://doi.org/10.1016/j.cub.2018.07.005	
45	Drosophila yakuba x D. santomea	diptera	https://doi.org/10.1111/j.0014-3820.2005.tb00972.x	
46	Caledia captiva (two chromosomal taxa)	grasshoppers	https://doi.org/10.1007/BF00327563	Hered-79, Chromosoma-79, Hered-88, Hered-80, HybZonEvoProcess-93, Evo-91, Hered-85, PNAS-87, CanJZool-90,
47	Chorthippus albomarginatus x C. oschei	grasshoppers	https://doi.org/10.1007/s00265-003-0595-2	BJLS-11,
48	Chorthippus brunneus x C. jacobsi	grasshoppers	https://doi.org/10.1111/j.0014-3820.2001.tb00832.x	Evo-02, JEvoBio-04, EcolEntom-03, PLOSOne-12,
49	Chorthippus parallelus (chromosomal races, Alps)	grasshoppers	J. EVOL. BIOL. 1 2 (1999) 577-585	
50	Chorthippus parallelus parallelus x C.p. erythropus	grasshoppers	https://doi.org/10.7717/peerj.1479	ChromRes, JEvolBiol, Evo_94, ProcB-05, JEvBiol-89, BJLS-85, JEvoBiol-91, PhilTransB-91, Hered-10, BJLS-85, Hered-93, JOrthoptRes-10, ProcB-94, JEvBiol-86, JEvBiol-07, BJLS-03, Genome-88, Hered-94, BJLS-92, EvolEcol-07,
51	Dichroplus pratensis chromosomal races	grasshoppers	https://doi.org/10.1038/hdy.1991.93	JEvBiol-04, JHered-11,
52	Melanoplus sanguinipes x M. devastator	grasshoppers	https://doi.org/10.1111/j.1558-5646.1996.tb03880.x	
53	Podisma pedestris (two chromosome races)	grasshoppers	https://doi.org/10.1038/hdy.1983.15	Hered-75, CanJZ-90, Hered-89, Hered-87, BJLS-08, Evo-08,
54	Stenobothrus rubicundus x S. clavatus	grasshoppers	https://doi.org/10.1002/ece3.2265	BioJLS, BJLS-12,
55	Vandiemenella viatica (two chromosomal races)	grasshoppers	https://doi.org/10.1111/j.1558-5646.2008.00526.x	
56	Carbula humerigera x C. putoni	hemiptera	https://doi.org/10.1111/een.12486	
57	Gerris costae costae x G. c. fieberi	hemiptera	https://doi.org/10.1046/j.1420-9101.1994.7060697.x	
58	Limnoporus dissortis x L. notabilis	hemiptera	https://doi.org/10.1111/j.1558-5646.1991.tb02642.x	CanJZoo-05, JEvoBiol-00,
59	Triatoma brasiliensis brasiliensis x Triatoma juazeirensis	hemiptera	https://doi.org/10.1016/j.meegid.2015.10.025	

60	Triatoma dimidiata genotypes	hemiptera	https://doi.org/10.1016/j.meegid.2009.09.009	
61	Operophtera brumata x O. bruceata	moths	https://doi.org/10.1111/mec.16349	
62	Thaumetopoea pityocampa x T. wilkinsoni	moths	https://doi.org/10.1002/ece3.6018	
63	Hemideina maori (two mitochondrial genotypes)	orthoptera	https://doi.org/10.1111/j.1365-294X.1996.tb00350.x	JRoySocNZea-03,
64	Hemideina thoracica (two chromosomal races)	orthoptera	https://doi.org/10.1046/j.1365-2540.2000.00796.x	
65	Bacillus rossius rossius x B. r. redtenbacheri	stick insect	https://doi.org/10.1673/031.010.14107	
66	Zootermopsis nevadensis nuttingi x Z. n. nevadensis	termites	https://doi.org/10.1007/s00040-009-0041-1	

OTHER INVERTEBRATES

1	Balanus glandula (two lineages)	barnacles	https://doi.org/10.1111/jbi.14142	
2	Macoma balthica x M. b. balthica	bivalves	https://doi.org/10.1111/j.1365-294X.2012.05586.x	
3	Menippe mercenaria x M. adina	crabs	Mar. Ecol. Prog. Ser. 52: 235-244, 1989	
4	Procambarus crayfish (two undescribed spp)	crayfishes	https://doi.org/10.1046/j.1420-9101.1992.5040643.x	
5	Gagrellopsis nodulifera chromosomal races	harvestmen	https://doi.org/10.1111/j.0014-3820.2000.tb00018.x	ZoolSci-91,
6	Eunicea flexuosa (two lineages)	octocorals	https://doi.org/10.1086/678403	
7	Palaemonetes kadiakensis populations	shrimps	https://doi.org/10.1111/j.1558-5646.1994.tb01318.x	
8	Paratya australiensis (two mtDNA lineages)	shrimps	https://doi.org/10.1093/jhered/esw033	
9	Ainohelix editha	snails	https://doi.org/10.1046/j.1365-294X.2003.01862.x	
10	Albinaria hippolyti aphrodite x A. h. harmonia	snails	https://doi.org/10.1111/j.1095-8312.1995.tb01027.x	ProcB-99,
11	Brephulopsis cylindrica x B.bidens	snails	Natura Montenegrina, 12, 3-4. 2013	
12	Cerion moralesi x C. Geophilus	snails	https://doi.org/10.2307/2407793	
13	Elimia livescens x E. virginica	snails	American Malacological Bulletin 11.1 (1994): 73-78.	
14	Littorina saxatilis ecotypes	snails	https://doi.org/10.1111/mec.14972	CurrZool, EvolEcol, JMolluscStud, JEvBiol-01, Evo-04, JEvoBiol-99, EvolEco-01, JEvBiol-04, EvolEcol-03,
15	Mandarina mandarina x M. chichijimana	snails	https://doi.org/10.1111/j.0014-3820.2005.tb01820.x	BJLS-08,
16	Radix balthica and an undescribed lineage	snails	https://doi.org/10.1111/mec.13049	
17	Delena cancerides (chromosomal races)	spiders	https://doi.org/10.1071/ZO9950173	
18	Lycosa ammophila x L. ericeticola	spiders	Revue Suisse de Zoologie, 1, 543-554 (1996)	
