# Supplementary material for: The puzzle of plant hybridisation: a high propensity to hybridise but few hybrid zones reported
Source: Heredity (Edinb). 2023 Oct 27;131(5-6):307–15. doi: 10.1038/s41437-023-00654-1 (PMC10673867; doi:10.1038/s41437-023-00654-1)
Supplement: Supplementary file 3 — Supplemental Table 3 [file 41437_2023_654_MOESM3_ESM.pdf]

Table S3.—Phylogenetic relatedness between taxa involved in 137 plant hybrid zones compiled in Abbott's (2017) review: non-sister species (brown); sister species (including subspecies of the same species, deep green); species reported as closely-related even if no explicit phylogeny was available, and those falling in the same polytomy (light green); insufficient or unavailable information (white).

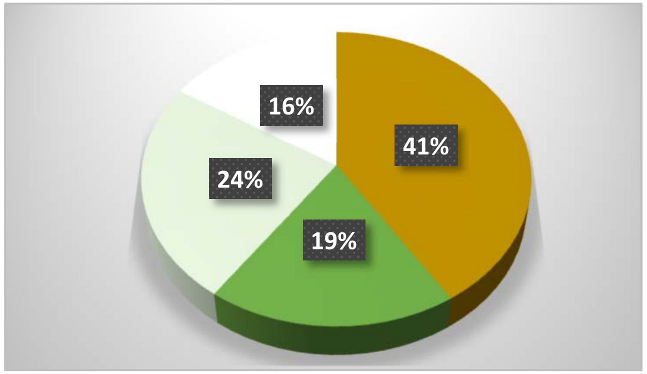

**non-sister species**	**56**	**40.88 %**
**sister species (including subspecies of the same species);**	**26**	**18.98 %**
**reported as closely-related even if no explicit phylogeny available, also including species in the same polytomy**	**33**	**24.09 %**
**insufficient or unavailable information**	**22**	**16.06 %**
	**137**	

	Taxa	Sister species?	Comments	Source for phylogenetic relationships	Source for the hybrid zone
1	*Abies alba* × *A. cephalonica* (Trees)	possibly		https://doi.org/10.1134/S1022795414010104	
2	*Abies homolepis* × *A. veitchii* (Trees)	No		https://doi.org/10.1016/j.ympev.2014.10.008	
3	*Aegilops geniculata* × *A. triuncialis* (Herbs)	No			
4	*Aesculus flava, A. pavia, A. sylvatica* (Trees)	No		https://doi.org/10.1002/tax.581012	
5	*Ainsliaea apiculata* × *A. faurieana* (Herbs)	?	faurieana does not appear in phylogenies		
6	*Alnus crispa* x *A. sinuata* (Trees)	possibly	polytomy	https://www.journals.uchicago.edu/doi/full/10.1086/382795	
7	*Anacamptis morio* × *A. papilionacea* (Herbs)	No		https://doi.org/10.1006/mpev.1999.0628	
8	*Antirrhinum majus. pseudomajus* (magenta flowers) × *A. m. striatum* (yellow) (Herbs)	yes	subspecies of the same sp		
9	*Aquilegia formosa* × *A. pubescens* (Herbs)	No		https://doi.org/10.1111/nph.12163	
10	*Aquilegia japonica* × *A. oxysepala* (Herbs)	yes		https://doi.org/10.1002/aps3.11412	
11	*Arctium lappa* × *A. minus* (Herbs)	polytomy	polytomy	https://doi.org/10.1002/tax.581016	https://doi.org/10.1007/s00606-007-0547-9

12	*Arctium lappa* × *A. tomentosum* (Herbs)	No	same section Arctium		https://doi.org/10.1007/s00606-007-0547-9
13	*Arctostaphylos patula* × *A. viscida* (Shrubs)	No		https://www.jstor.org/stable/41425263	
14	*Argyranthemum formosa* × *A. pubescens* (Herbs)	No			
15	*Artemisia tridentata* ssp. *tridentata* × *A. t.* ssp. *vaseyana* (Shrubs)	possibly	subspecies…		
16	*Asclepias exaltata* × *A. syriaca* (Herbs)	No		https://doi.org/10.1002/ajb2.1062	
17	*Banksia hookeriana* × *B. prionotes* (Shrubs)	yes		https://doi.org/10.1111/j.1469-8137.2011.03663.x	
18	*Banksia oblongifolia* × *B. robur* (Shrubs)	yes		https://doi.org/10.1111/j.1469-8137.2011.03663.x	
19	*Begonia heracleifolia* × *B. nelumbiifolia* (Herbs)	?			
20	*Begonia heracleifolia* × *B* *sericoneura* (Herbs)	?			
21	*Betula alleghaniensis* × *B. papyrifera* (Trees)	No	Not based on nrDNA (1st doi); but possibly based on second; close anyway	https://doi.org/10.3732/ajb.91.11.1834https://www.jstor.org/stable/23314506	https://doi.org/10.1007/s11295-015-0922-6
22	*Betula ermanii* × *B. pubescens* (Trees)	No	"Järvinen et al. (2004) pointed out the similarities between B. pubescens and B. ermanii which could support Walters' (1968) idea that they shared at least one common ancestor. " both tetraploids		https://doi.org/10.1111/mec.13885

23	*Betula pendula* × *B. plathyphylla* (Trees)	No	"Betula pubescens is an allotetraploid and one of the parental species was Betula pendula" "Betula platyphylla and B. pendula are close relatives in both molecular phylogenies and chemotaxonomy (e.g. Keinänen et al. 1999; Järvinen et al. 2004; Li et al. 2007)," They're both diploids		https://doi.org/10.1111/mec.13885
24	*Borrichia arborescens* × *B. frutescens* (Shrubs)	?	Both members of a 6-species genus		https://doi.org/10.3732/ajb.91.11.1757
25	*Callicarpa japonica* × *C. mollis* (Shrubs/Trees)	No	Both in sect. Verticirima, based on their stamen characters, but morphologically distinct. No phylog. avail.		https://doi.org/10.1046/j.1365-294X.2003.01961.x
26	*Carex curvula curvula* × *C. c. rosae* (Herbs)	yes	subspecies of the same sp		https://doi.org/10.1073/pnas.2237235100
27	*Carex limosa* × *C. rariflora* (Herbs)	No	Not in 1st doi, but both Sect. Limosae, and rariflora sister to a pair including limosa	https://doi.org/10.1007/s00606-004-0128-0	https://doi.org/10.3732/ajb.89.4.642
28	*Ceanothus roderickii* × *C. cuneatus* (Shrubs)	closely-related	"very closely related; phylogenetic analyses suggest that C. roderickii is derived from within C. cuneatus (Burge and Manos, 2011)"		https://doi.org/10.3732/ajb.1200604
29	*Cirsium californicum* × *C. occidentale* (Herbs)	No	belong in different taxonomic series. No phylogeny avail.		https://www.jstor.org/stable/41426184
30	*Clarkia xantiana parviflora* × *C. x. xantiana* (Herbs)	yes	subspecies of the same sp		https://doi.org/10.1111/evo.12488
31	*Costus pulverulentus* × *C. scaber* (Herbs)	polytomy	"closely related species ... both part of an unresolved clade together with other closely related Central American species" "two members of a rapid Neotropical species radiation"		https://doi.org/10.1111/mec.12442
32	*Dubautia arborea* × *D. ciliolata* (Shrubs)	polytomy	"Dubautia arborea and Dubautia ciliolata represent a species complex in the Hawaiian silversword alliance"		https://doi.org/10.1111/j.1365-294X.2007.03447.x

33	*Dubautia ciliolata* × *D. scabra* (Shrubs)	polytomy	Apparently not, but close and part o the same Silversword adaptive radiation characterized by interbreeding among morphologically distinct spp		https://doi.org/10.2307/3558414
34	*Eleocharis cellulosa x E. interstincta* (Herbs)	No	"... more distant taxa (like the pair E. cellulosa–E. interstincta in our study)".		https://doi.org/10.3732/ajb.1000029
35	*Epidendrum calanthum* × *E. cochlidium* (Herbs)	No	The three species belong to the subgenus *Amphiglottium* and are not closely related since *E. calanthum* and *E. cochlidium* belong to two different phylogenetic groups		https://doi.org/10.1371/journal.pone.0080662
36	*Epidendrum calanthum* × *E. schistochilum* (Herbs)	No	The three species belong to the subgenus *Amphiglottium* and are not closely related since *E. calanthum* and *E. cochlidium* belong to two different phylogenetic groups		https://doi.org/10.1371/journal.pone.0080662
37	*Epidendrum cochlidium* × *E. schistochilum* (Herbs)	No	The three species belong to the subgenus *Amphiglottium* and are not closely related since *E. calanthum* and *E. cochlidium* belong to two different phylogenetic groups		https://doi.org/10.1371/journal.pone.0080662
38	*Eucalyptus acmenoides* × *E. cloeziana* (Trees)	No	cloeziana sister to subgen. Monocalyptus where acmenoides belongs		https://doi.org/10.1006/anbo.2001.1507
39	*Eucalyptus aggregata* × *E. rubida* (Trees)	No	"aggregata assigned to series Faveolatae and E. rubida to series Viminales"		https://doi.org/10.1038/hdy.2010.127
40	*Eucalyptus brownii* × *E. populnea* (Trees)	?	?		https://doi.org/10.1046/j.1365-294X.2003.01970.x
41	*E. cordata x E. globulus* (Trees)	closely-related	"closely related"	https://doi.org/10.1016/j.ympev.2011.02.003	
42	*Fraxinus angustifolia* × *F. excelsior* (Trees)	yes	in 1st doi; "closely related" in 2nd doi	https://doi.org/10.1371/journal.pone.0080431	https://doi.org/10.1186/1471-2148-6-96

43	*Gaillardia. pulchella* , calcicole × calcifuge (Herbs)	yes	even more than sister (ecotypes of same species)		https://doi.org/10.1093/oxfordjournals.jhered.a110205
44	*Geum rivale* (outcrosser) × *G. urbanum* (selfer) - (Herbs)	yes	in first doi; "closely related" in 2nd doi	https://doi.org/10.1043/0363-6445-27.2.303	https://doi.org/10.1038/hdy.2011.9
45	*Gliricidia maculata* × *G. sepium* (Trees)	possibly	for ITS, not for matK	https://www.jstor.org/stable/3094008	
46	*Helianthus annuus* × *H. petiolaris* (Herbs)	No		https://doi.org/10.3732/ajb.94.11.1837	
47	*Helianthus annuus* × *H. bolanderi* (Herbs)	No		https://doi.org/10.3732/ajb.94.11.1837	
48	*Impatiens javensis* × *I. radicans* (Herbs)	?	???		https://doi.org/10.3732/ajb.91.12.2119
49	*Ipomopsis aggregata* × *I. tenuituba* (Herbs)	yes	"These are sister species"		https://doi.org/10.1111/j.1558-5646.2008.00460.x
50	*Ipomopsis aggregata* subsp. *candida* × subsp. *collina* (Herbs)	yes	subspecies of the same sp		https://doi.org/10.1111/mec.13752
51	*Iris brevicaulis* × *I. fulva* (Herbs)	yes	two of the members of the "Louisiana iris" species complex	https://doi.org/10.1111/bij.12884	https://doi.org/10.1002/ece3.964
52	*Iris fulva* × *I. hexagona* (Herbs)	yes	two of the members of the "Louisiana iris" species complex	https://doi.org/10.1111/bij.12884	https://doi.org/10.1002/j.1537-2197.1994.tb15611.x
53	*Leucosceptrum japonicum* × *L. stellipilum* (Shrubs/Small trees)	?	not stated, the genus only includes 5 spp		https://doi.org/10.1371/journal.pone.0116411
54	*Liparis kumokiri* (self-compatible) × *L. makinoana* (self-incompatible) (Herbs)	No		https://doi.org/10.1007/s10265-007-0077-0	https://doi.org/10.1111/j.1365-294X.2005.02738.x
55	*Lomatia myricoides* × *L. silaifolia* (Shrubs)	No	"Although closely related, they are not sister species"		https://doi.org/10.1093/aob/mct314

56	*Magnolia salicifolia* × *M. stellata* (Trees)	?	same subgen. Yulania	https://doi.org/10.1007/PL0001388	
57	*Metrosideros polymorpha* high × low altitude populations (Trees)	yes	even more than sister (ecotypes of same species)		https://doi.org/10.1111/mec.14016
58	*Mimulus guttatus* (outcrosser) × *M. nasutus* (selfer) - (Herbs)	closely	closely related		https://doi.org/10.1111/mec.13630
59	*Mimulus aurantiacus* ssp. *australis (yellow flowered)* × ssp. *puniceus* (red flowered) (Herbs)	?	cf. Fig. 3. Same section anyway (Diplacus)	https://doi.org/10.3732/ajb.1700234	
60	*Ophrys fusca* × *O. lutea* (Herbs)	?	close in section Pseudophrys		https://doi.org/10.3732/ajb.1500252
61	*Orchis mascula* × *O. pauciflora* (Herbs)	No	but close, paucif. sister to a mascula-provincialis pair	https://doi.org/10.1006/mpev.1999.0628	
62	*Orchis militaris* × *O. purpurea* (Herbs)	polytomy	In a 3-taxon polytomy in 1st doi; 'closely related' in 2nd doi	https://doi.org/10.1006/mpev.1999.0628	https://doi.org/10.1186/1471-2148-12-178
63	*Pericallis cruenta* × *P. echinata* (Herbs)	polytomy	in a 5-taxon polytomy	https://doi.org/10.2307/3647452	https://doi.org/10.1007/s00606-012-0624-6
64	*Pericallis cruenta* × *P. tussilaginus* (Herbs)	No		https://doi.org/10.2307/3647452	https://doi.org/10.1007/s00606-012-0624-6
65	*Phlomis crinita* × *P. lychnitis* (Herbs)	closely-related	closely related anyway	https://doi.org/10.1007/s12225-011-9257-0	
66	*Phlox cuspidata* (Pink flower, SC) × *P. drummondii* (Red flower, SI) (Herbs)	closely-related	although possibly with roemeriana in between; 'closely related' (2nd doi)	https://doi.org/10.3732/ajb.89.8.1324	DOI: 10.1126/science.1215198
67	*Phyllodace aleutica* × *P. caerulea* (Shrubby herbs)	?			https://doi.org/10.1111/j.1442-1984.2010.00301.x

68	*Picea abies* × *P. obovata* (Trees)	yes	sister species in the 2nd doi	https://doi.org/10.1016/j.ympev.2013.07.004	https://doi.org/10.1111/mec.13654
69	*Picea engelmanii* × *P. glauca* (Trees)	yes		https://doi.org/10.1016/j.ympev.2013.07.004	
70	*Picea glauca* × *P. sitchensis* (Trees)	No	but close: sitchensis sister to the pair engelmannii - glauca	https://doi.org/10.1016/j.ympev.2013.07.004	
71	*P. mariana* × *P. rubens* (Trees)	yes		https://doi.org/10.1016/j.ympev.2013.07.004	
72	*Pinus banksiana* × *P. contorta* (Trees)	polytomy	same polytomy including 4 spp	https://doi.org/10.2307/25065300	
73	*Pinus echinata* × *P. taeda* (Trees)	No	but same subsect Australes in Sect. Trifoliae, possibly a polytomy	https://doi.org/10.2307/25065300	
74	*Pinus hwangshanensis* × *P. massoniana* (Trees)	No	closely related in 2nd doi	https://doi.org/10.2307/25065300	https://doi.org/10.1371/journal.pone.0101106
75	*Pinus. mugo* × *P. sylvestris* (complex) - (Trees)	No	but same sect. Pinus	https://doi.org/10.2307/25065300	
76	*Pinus parviflora* × *P. pumila* (Trees)	No	but same sect. Quinquefoliae and Subsect. Strobus	https://doi.org/10.2307/25065300	
77	*Piriqueta caroliniana caroliana* × *P. c. viridis* (Herbs)	Probably	called morphotypes of a 'caroliniana' complex	https://doi.org/10.1111/j.1558-5646.1999.tb04519.x	
78	*Pitcairnia albiflos* × *P. staminea* (Herbs)	closely-related	closely related	https://doi.org/10.1111/j.1365-294X.2011.05143.x	
79	*Platanthera aquilonis* × *P. dilatata* (Herbs)	No		https://doi.org/10.1016/j.ympev.2021.107070	
80	*Polystichum imbricans* × *P. munitum* (Herbs – ferns)	closely-related	"closely related" (1st doi) same polytomy (2nd)	https://doi.org/10.3732/ajb.90.3.508	
81	*Populus alba* × *P. tremula* (Trees)	polytomy	related: same polytomy	https://doi.org/10.3732/ajb.91.9.1398	

82	*Populus angustifolia x P.deltoides* (Trees)	No		https://doi.org/10.3732/ajb.91.9.1398	
83	*Populus angustifolia × P. fremontii* (Trees)	No		https://doi.org/10.3732/ajb.91.9.1398	
84	*Populus balsamifera × P. deltoides* (Trees)	No		https://doi.org/10.3732/ajb.91.9.1398	
85	*Populus laurifolia × P. nigra* (Trees)	No	"distantly related spp" (2nd doi)	https://doi.org/10.3732/ajb.91.9.1398	https://doi.org/10.1186/s12870-016-0776-6
86	*Primula beesiana × P. bulleyana* (Herbs)	No	but members of subg. Aleuritia sect. Proliferae	https://doi.org/10.1134/S1022795409060052	
87	*Quercus affinis × Q. laurina* (Trees)	NO		https://doi.org/10.3390/f12060786	
88	*Quercus austroco-chinchinensis × Q. kerrii* (Trees)	closely-related	"closely-related species"	https://doi.org/10.3389/fpls.2017.00229	
89	*Quercus berberidifolia × Q. durata* (Trees)	yes		https://doi.org/10.3390/f12060786	
90	*Quercus coccifera × Q. ilex* (Trees)	No	but same "ilex group"	https://doi.org/10.1006/mpev.1999.0614	
91	*Quercus crassifolia × Q. crassipes* (Trees)	No		https://doi.org/10.3390/f12060786	
92	*Quercus crispula × Q. dentata* (Trees)	closely-related	closely related species	https://doi.org/10.1007/s10265-018-01079-2	
93	*Quercus douglasii x Q. lobata* (Trees)	closely-related	closely related species	https://doi.org/10.3732/ajb.89.11.1792	
94	*Quercus gambelii × Q. grisea* (Trees)		different subsections of white oaks	https://doi.org/10.1111/j.1558-5646.1997.tb03658.x	
95	*Quercus geminata × Q. virginiana* (Trees)	yes	"sister oak species" in the title	https://doi.org/10.3732/ajb.0800315	

96	*Quercus ilex* × *Q. suber* (Trees)	No	although same section, different "groups"	https://doi.org/10.1006/mpev.1999.0614
97	*Quercus kelloggii* × *Q. wislizen* i (Trees)	?	all belong in Q. subsect. Agrifoliae	https://doi.org/10.3390/f12060786
98	*Quercus liaotungensis* × *Q. mongolica* (Trees)	closely-related	"closely related in the title"	https://doi.org/10.1371/journal.pone.0015529
99	*Quercus magnoliifolia* × *Q. resinosa* (Trees)	?	both in section Quercus	https://doi.org/10.1086/650317
100	*Quercus petraea* × *Q. pubescens* (Trees)	No		https://doi.org/10.1080/14772000.2014.941037
101	*Quercus petraea* × *Q. pyrenaica* (Trees)	No		https://doi.org/10.1080/14772000.2014.941037
102	*Quercus petraea* × *Q. robur* (Trees)	yes		https://doi.org/10.1080/14772000.2014.941037
103	Mixed stand of *Quercus frainetto, Q. petraea, Q. pubescens, Q. robur* (Trees)	?	robur and petraea, pubescens sister. All subsect. Quercus	https://doi.org/10.1080/14772000.2014.941037
104	Hybridization between *Quercus coccinea, Q. falcata, Q. rubra* , *Q. velutina* (Trees)	?	all except falcata (Subsect. Phellos) belong in subsect. Coccineae	https://doi.org/10.3390/f12060786
105	Hybridization between *Quercus ellipsoidalis, Q. velutina, Q. coccinea, Q. rubra* (Trees)	?	all belong in Q. subsect. Coccineae	https://doi.org/10.3390/f12060786
106	Hybridization of *Quercus wislizeni* with *Q. agrifolia, Q. kelloggii* , *Q. parvula* (Trees)	?	all belong in Q. subsect. Agrifoliae	https://doi.org/10.3390/f12060786
107	*Rhinanthus angustifolia* × *R. minor* (Herbs)	closely-related	closely related species	https://doi.org/10.1002/ece3.276
108	*Rhizophora apiculata* × *R. mucronata* (Trees)	No		https://doi.org/10.1371/journal.pone.0145058

109	*Rhizophora apiculata* × *R. stylosa* (Trees)	No		https://doi.org/10.1371/journal.pone.0145058
110	*Rhizophora mangle* × *R. racemosa* (Trees)	yes		https://doi.org/10.1371/journal.pone.0145058
111	*Rhizophroa samoensis* × *R. stylosa* (Trees)	NO	samoensis = mangle	https://doi.org/10.1371/journal.pone.0145058
112	*Rhododendron aganniphum* × *R. phaeochrysum* (Shrubs)	possibly	"part of a particularly complex group of species within subsection Taliensis of subgenus Hymenanthes (Chamberlain 1982)."	https://doi.org/10.1002/ece3.1570
113	*Rhododendron caucasicum* × *R. ponticum* (Shrubs)	?	"species of Rhododendron section Hymenanthes, these species are highly interfertile"	https://doi.org/10.1046/j.1365-294X.2003.01942.x
114	*Rhododendron decorum* × *R. delavayi* (Shrubs)	No	"distantly related (in title)" "belong to different subsections of subgenus Hymenanthes,"	https://doi.org/10.1111/j.1095-8339.2007.00752.x
115	*Rhododendron delavayi* × *R. irroratum* (Shrubs)	No	"Rhododendron delavayi, R. decorum and R. irroratum are members of subsections Arborea, Fortunea and Irrorata, respectively (Chamberlain,	https://doi.org/10.1093/aob/mcp267
116	*Rhododendron eriocarpum* × *R. indicum* (Shrubs)	closely-related	"The two are closely related species in the Tsutsuji series "	https://doi.org/10.1007/s10265-008-0167-7
117	*Rhododendron ferrugineum* × *R. hirsutum* (Shrubs)	closely-related	closely related, same subsection Rhododendron	https://en.wikipedia.org/wiki/Rhododendron_hirsutum
118	*Rhododendron spiciferum* x *R. spinuliferum* (Shrubs)	?	belong to subsection Scabrifolia, sect. Rhododendron of subgenus Rhododendron, a subsection that consists of eight species endemic to southwest China	https://doi.org/10.1111/j.1759-6831.2012.00243.x
119	*Rorippa amphibia* (self-incompatible) × *R. palustris* (self-compatible) - (Herbs)	polytomy	polytomy	https://onlinelibrary.wiley.com/doi/pdf/10.1055/s-2002-20442
120	*Rorippa amphibia* × *R. sylvestris* (Herbs)	?	different accessions in different positions	https://onlinelibrary.wiley.com/doi/pdf/10.1055/s-2002-20442

121	*Sabatia arenicola* × *S. formosa* (Herbs)	?	"two members of section Campestria"	https://doi.org/10.2307/2418534
122	*Salix alba* × *S. fragilis* (Trees)	No	but closely related anyway	https://doi.org/10.1007/BF00984656
123	*Salix eriocephala* × *S. sericea* (Small trees)	?	not reported	
124	*Salix helvetica* × *S. purpurea* (Shrubs/Dwarf trees)	No	"he two species are morphologically distinct and belong to two different, unrelated sections within the genus (Skvortsov, 1999)"	https://doi.org/10.1038/hdy.2016.14
125	Mixed stand of *Salix dasyclados, S. schwerinii* and *S. viminalis* (Trees)	closely-related	closely related	https://doi.org/10.1186/s12862-015-0461-7
126	Mixed stand of *Sarracenia leucophylla, S. alata* , and *S. rubra* (Herbs)	closely-related	Based on the phylogeny. But closely related anyway	https://doi.org/10.2307/2419743
127	*Schiedea menziesii* (hermaphroditic) × *S. salicaria* (gynodioecious) (Shrubs)	No		https://doi.org/10.2307/2419665
128	*Senecio aethnensis* × *S. chrysanthemifolius* (Herbs)	possibly not	aethnensis sister to an unresolved Med. Clade where chrysanthemifolius is both with low support	https://doi.org/10.1111/j.0014-3820.2001.tb01312.x
129	*Senecio hercynicus* × *S. ovatus* (Herbs)	possibly	losely knit group of species (syngameon)	https://doi.org/10.1007/s00606-010-0295-0
130	*Silene dioica* × *S. latifolia* (Herbs)	yes		https://doi.org/10.1111/j.1365-294X.2008.03709.x
131	*Sphagnum capillifolium* × *S. quinquefarium* (Herbs, mosses)	No		https://www.jstor.org/stable/3244158
132	*Tithonia rotundifolia* × *T. tubaeformis* (Herbs)	No		https://doi.org/10.1111/j.0014-3820.2000.tb00050.x
133	*Vincetoxicum atratum* × *V. japonicum* (Herbs)	No		https://doi.org/10.1111/j.1095-8312.2007.00896.x
134	*Viola bissetii* × *V. rossii* (Herbs)	yes		

135	*Viola chaeropphylloides* × *V. eizanensis* (Herbs)	yes	https://doi.org/10.1007/s10265-009-0235-7
136	*Yucca brevifolia* × *Y. jaegeriana* (Trees)	yes	https://doi.org/10.1002/ajb2.1633
137	*Z aluzianskya microsiphon* × *Z. natalensis* (Herbs)	No	https://doi.org/10.1600/0363644053661977
			https://doi.org/10.1016/j.ympev.2017.04.011
