# Supplementary material for: The puzzle of plant hybridisation: a high propensity to hybridise but few hybrid zones reported
Source: Heredity (Edinb). 2023 Oct 27;131(5-6):307–15. doi: 10.1038/s41437-023-00654-1 (PMC10673867; doi:10.1038/s41437-023-00654-1)
Supplement: Supplementary file 5 — Supplemental Table 5 [file 41437_2023_654_MOESM5_ESM.pdf]

Table S5.—Life spans of taxa involved in 137 plant hybrid zones compiled in Abbott's (2017) review.

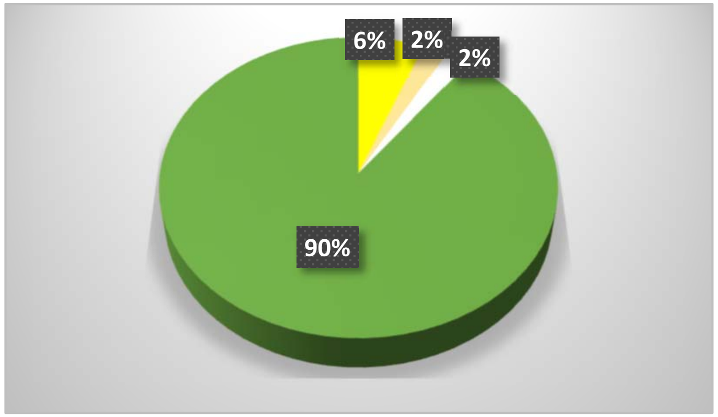

**annual**	**8**	**5.84 %**
**biennial**	**3**	**2.19 %**
**biennial/perennial or usually perennial**	**3**	**2.19 %**
**perennial**	**123**	**89.78 %**
**total**	**137**	

	Hybrid zone	Life form	Life span
1	*Abies alba* × *A. cephalonica* (Trees)	Trees	perennial
2	*Abies homolepis* × *A. veitchii* (Trees)	Trees	perennial
3	*Aegilops geniculata* × *A. triuncialis* (Herbs)	Herbs	annual
4	*Aesculus flava, A. pavia, A. sylvatica* (Trees)	Trees	perennial
5	*Ainsliaea apiculata* × *A. faurieana* (Herbs)	Herbs	perennial
6	*Alnus crispa x A. sinuata* (Trees)	Trees	perennial
7	*Anacamptis morio* × *A. papilionacea* (Herbs)	Herbs	perennial
8	*Antirrhinum majus. pseudomajus* (magenta flowers) × *A. m. striatum* (yellow) (Herbs)	Herbs	biennial/perennial
9	*Aquilegia formosa* × *A. pubescens* (Herbs)	Herbs	perennial
10	*Aquilegia japonica* × *A. oxysepala* (Herbs)	Herbs	perennial
11	*Arctium lappa* × *A. minus* (Herbs)	Herbs	biennial

12	*Arctium lappa* × *A. tomentosum* (Herbs)	Herbs	biennial
13	*Arctostaphylos patula* × *A. viscida* (Shrubs)	Shrubs	perennial
14	*Argyranthemum formosa* × *A. pubescens* (Herbs)	Herbs	perennial
15	*Artemisia tridentata* ssp. *tridentata* × *A. t.* ssp. *vaseyana* (Shrubs)	Shrubs	perennial
16	*Asclepias exaltata* × *A. syriaca* (Herbs)	Herbs	perennial
17	*Banksia hookeriana* × *B. prionotes* (Shrubs)	Shrubs	perennial
18	*Banksia oblongifolia* × *B. robur* (Shrubs)	Shrubs	perennial
19	*Begonia heracleifolia* × *B. nelumbiifolia* (Herbs)	Herbs	perennial
20	*Begonia heracleifolia* × *B sericoneura* (Herbs)	Herbs	perennial
21	*Betula alleghaniensis* × *B. papyrifera* (Trees)	Trees	perennial
22	*Betula ermanii* × *B. pubescens* (Trees)	Trees	perennial
23	*Betula pendula* × *B. plathyphylla* (Trees)	Trees	perennial
24	*Borrichia arborescens* × *B. frutescens* (Shrubs)	Shrubs	perennial
25	*Callicarpa japonica* × *C. mollis* (Shrubs/Trees)	Shrubs/Trees	perennial
26	*Carex curvula curvula* × *C. c. rosae* (Herbs)	Herbs	perennial
27	*Carex limosa* × *C. rariflora* (Herbs)	Herbs	perennial
28	*Ceanothus roderickii* × *C. cuneatus* (Shrubs)	Shrubs	perennial
29	*Cirsium californicum* × *C. occidentale* (Herbs)	Herbs	biennial/perennial
30	*Clarkia xantiana parviflora* × *C. x. xantiana* (Herbs)	Herbs	annual
31	*Costus pulverulentus* × *C. scaber* (Herbs)	Herbs	perennial
32	*Dubautia arborea* × *D. ciliolata* (Shrubs)	Shrubs	perennial
33	*Dubautia ciliolata* × *D. scabra* (Shrubs)	Shrubs	perennial
34	*Eleocharis cellulosa x E. interstincta* (Herbs)	Herbs	perennial
35	*Epidendrum calanthum* × *E. cochlidium* (Herbs)	Herbs	perennial
36	*Epidendrum calanthum* × *E. schistochilum* (Herbs)	Herbs	perennial
37	*Epidendrum cochlidium* × *E. schistochilum* (Herbs)	Herbs	perennial
38	*Eucalyptus acmenoides* × *E. cloeziana* (Trees)	Trees	perennial
39	*Eucalyptus aggregata* × *E. rubida* (Trees)	Trees	perennial
40	*Eucalyptus brownii* × *E. populnea* (Trees)	Trees	perennial
41	*E. cordata x E. globulus* (Trees)	Trees	perennial
42	*Fraxinus angustifolia* × *F. excelsior* (Trees)	Trees	perennial
43	*Gaillardia pulchella* , calcicole × calcifuge (Herbs)	Herbs	perennial
44	*Geum rivale* (outcrosser) × *G. urbanum* (selfer) - (Herbs)	Herbs	perennial

45	*Gliricidia maculata* × *G. sepium* (Trees)	Trees	perennial
46	*Helianthus annuus* × *H. petiolaris* (Herbs)	Herbs	annual
47	*Helianthus annuus* × *H. bolanderi* (Herbs)	Herbs	annual
48	*Impatiens javensis* × *I. radicans* (Herbs)	Herbs	perennial
49	*Ipomopsis aggregata* × *I. tenuituba* (Herbs)	Herbs	perennial
50	*Ipomopsis aggregata* subsp. *candida* × subsp. *collina* (Herbs)	Herbs	perennial
51	*Iris brevicaulis* × *I. fulva* (Herbs)	Herbs	perennial
52	*Iris fulva* × *I. hexagona* (Herbs)	Herbs	perennial
53	*Leucosceptrum japonicum* × *L. stellipilum* (Shrubs/Small trees)	Shrubs/Small trees	perennial
54	*Liparis kumokiri* (self-compatible) × *L. makinoana* (self-incompatible) (Herbs)	Herbs	perennial
55	*Lomatia myricoides* × *L. silaifolia* (Shrubs)	Shrubs	perennial
56	*Magnolia salicifolia* × *M. stellata* (Trees)	Trees	perennial
57	*Metrosideros polymorpha* high × low altitude populations (Trees)	Trees	perennial
58	*Mimulus guttatus* (outcrosser) × *M. nasutus* (selfer) - (Herbs)	Herbs	perennial
59	*Mimulus aurantiacus* ssp. *australis (yellow flowered)* × ssp. *puniceus* (red flowered) (Herbs)	Herbs	perennial
60	*Ophrys fusca* × *O. lutea* (Herbs)	Herbs	perennial
61	*Orchis mascula* × *O. pauciflora* (Herbs)	Herbs	perennial
62	*Orchis militaris* × *O. purpurea* (Herbs)	Herbs	perennial
63	*Pericallis cruenta* × *P. echinata* (Herbs)	Herbs	perennial
64	*Pericallis cruenta* × *P. tussilaginus* (Herbs)	Herbs	perennial
65	*Phlomis crinita* × *P. lychnitis* (Herbs)	Herbs	perennial
66	*Phlox cuspidata* (Pink flower, SC) × *P. drummondii* (Red flower, SI) (Herbs)	Herbs	annual
67	*Phyllodace aleutica* × *P. caerulea* (Shrubby herbs)	Shrubby Herbs	perennial
68	*Picea abies* × *P. obovata* (Trees)	Trees	perennial
69	*Picea engelmanii* × *P. glauca* (Trees)	Trees	perennial
70	*Picea glauca* × *P. sitchensis* (Trees)	Trees	perennial
71	*Picea mariana* × *P. rubens* (Trees)	Trees	perennial
72	*Pinus banksiana* × *P. contorta* (Trees)	Trees	perennial
73	*Pinus echinata* × *P. taeda* (Trees)	Trees	perennial
74	*Pinus hwangshanensis* × *P. massoniana* (Trees)	Trees	perennial
75	*Pinus. mugo* × *P. sylvestris* (complex) - (Trees)	Trees	perennial
76	*Pinus parviflora* × *P. pumila* (Trees)	Trees	perennial

77	*Piriqueta caroliniana caroliana* × *P. c. viridis* (Herbs)	Herbs	usually perennial
78	*Pitcairnia albiflos* × *P. staminea* (Herbs)	Herbs	perennial
79	*Platanthera aquilonis* × *P. dilatata* (Herbs)	Herbs	perennial
80	*Polystichum imbricans* × *P. munitum* (Herbs – ferns)	Herbs – ferns	perennial
81	*Populus alba* × *P. tremula* (Trees)	Trees	perennial
82	*Populus angustifolia x P.deltoides* (Trees)	Trees	perennial
83	*Populus angustifolia* × *P. fremontii* (Trees)	Trees	perennial
84	*Populus balsamifera* × *P. deltoides* (Trees)	Trees	perennial
85	*Populus laurifolia* × *P. nigra* (Trees)	Trees	perennial
86	*Primula beesiana* × *P. bulleyana* (Herbs)	Herbs	perennial
87	*Quercus affinis* × *Q. laurina* (Trees)	Trees	perennial
88	*Quercus austroco-chinchinensis* × *Q. kerrii* (Trees)	Trees	perennial
89	*Quercus berberidifolia* × *Q. durata* (Trees)	Trees	perennial
90	*Quercus coccifera* × *Q. ilex* (Trees)	Trees	perennial
91	*Quercus crassifolia* × *Q. crassipes* (Trees)	Trees	perennial
92	*Quercus crispula* × *Q. dentata* (Trees)	Trees	perennial
93	*Quercus douglasii x Q. lobata* (Trees)	Trees	perennial
94	*Quercus gambelii* × *Q. grisea* (Trees)	Trees	perennial
95	*Quercus geminata* × *Q. virginiana* (Trees)	Trees	perennial
96	*Quercus ilex* × *Q. suber* (Trees)	Trees	perennial
97	*Quercus kelloggii* × *Q. wislizen* i (Trees)	Trees	perennial
98	*Quercus liaotungensis* × *Q. mongolica* (Trees)	Trees	perennial
99	*Quercus magnoliifolia* × *Q. resinosa* (Trees)	Trees	perennial
100	*Quercus petraea* × *Q. pubescens* (Trees)	Trees	perennial
101	*Quercus petraea* × *Q. pyrenaica* (Trees)	Trees	perennial
102	*Quercus petraea* × *Q. robur* (Trees)	Trees	perennial
103	Mixed stand of *Quercus frainetto, Q. petraea, Q. pubescens, Q. robur* (Trees)	Trees	perennial
104	Hybridization between *Quercus coccinea, Q. falcata, Q. rubra* , *Q. velutina* (Trees)	Trees	perennial
105	Hybridization between *Quercus ellipsoidalis, Q. velutina, Q. coccinea, Q. rubra* (Trees)	Trees	perennial
106	Hybridization of *Quercus wislizeni* with *Q. agrifolia, Q. kelloggii* , *Q. parvula* (Trees)	Trees	perennial

107	*Rhinanthus angustifolia* × *R. minor* (Herbs)	Herbs	annual
108	*Rhizophora apiculata* × *R. mucronata* (Trees)	Trees	perennial
109	*Rhizophora apiculata* × *R. stylosa* (Trees)	Trees	perennial
110	*Rhizophora mangle* × *R. racemosa* (Trees)	Trees	perennial
111	*Rhizophroa samoensis* × *R. stylosa* (Trees)	Trees	perennial
112	*Rhododendron aganniphum* × *R. phaeochrysum* (Shrubs)	Shrubs	perennial
113	*Rhododendron caucasicum* × *R. ponticum* (Shrubs)	Shrubs	perennial
114	*Rhododendron decorum* × *R. delavayi* (Shrubs)	Shrubs	perennial
115	*Rhododendron delavayi* × *R. irroratum* (Shrubs)	Shrubs	perennial
116	*Rhododendron eriocarpum* × *R. indicum* (Shrubs)	Shrubs	perennial
117	*Rhododendron ferrugineum* × *R. hirsutum* (Shrubs)	Shrubs	perennial
118	*Rhododendron spiciferum x R. spinuliferum* (Shrubs)	Shrubs	perennial
119	*Rorippa amphibia* (self-incompatible) × *R. palustris* (self-compatible) - (Herbs)	Herbs	perennial
120	*Rorippa amphibia* × *R. sylvestris* (Herbs)	Herbs	perennial
121	*Sabatia arenicola* × *S. formosa* (Herbs)	Herbs	annual
122	*Salix alba* × *S. fragilis* (Trees)	Trees	perennial
123	*Salix eriocephala* × *S. sericea* (Small trees)	Small trees	perennial
124	*Salix helvetica* × *S. purpurea* (Shrubs/Dwarf trees)	Shrubs/Dwarf trees	perennial
125	Mixed stand of *Salix dasyclados, S. schwerinii* and *S. viminalis* (Trees)	Trees	perennial
126	Mixed stand of *Sarracenia leucophylla, S. alata*, and *S. rubra* (Herbs)	Herbs	perennial
127	*Schiedea menziesii* (hermaphroditic) × *S. salicaria* (gynodioecious) (Shrubs)	Shrubs	perennial
128	*Senecio aethnensis* × *S. chrysanthemifolius* (Herbs)	Herbs	perennial
129	*Senecio hercynicus* × *S. ovatus* (Herbs)	Herbs	perennial
130	*Silene dioica* × *S. latifolia* (Herbs)	Herbs	biennial
131	*Sphagnum capillifolium* × *S. quinquefarium* (Herbs, mosses)	Herbs, mosses	perennial
132	*Tithonia rotundifolia* × *T. tubaeformis* (Herbs)	Herbs	annual
133	*Vincetoxicum atratum* × *V. japonicum* (Herbs)	Herbs	perennial
134	*Viola bissetii* × *V. rossii* (Herbs)	Herbs	perennial
135	*Viola chaeropphylloides* × *V. eizanensis* (Herbs)	Herbs	perennial
136	*Yucca brevifolia* × *Y. jaegeriana* (Trees)	Trees	perennial
137	*Z aluzianskya microsiphon* × *Z. natalensis* (Herbs)	Herbs	perennial
