# Supplementary material for: The puzzle of plant hybridisation: a high propensity to hybridise but few hybrid zones reported
Source: Heredity (Edinb). 2023 Oct 27;131(5-6):307–15. doi: 10.1038/s41437-023-00654-1 (PMC10673867; doi:10.1038/s41437-023-00654-1)
Supplement: Supplementary file 6 — Supplemental Table 6 [file 41437_2023_654_MOESM6_ESM.pdf]

Table S6.—Results of a series of surveys in Clarivate Web of Science to compare the report of hybrid zones in animal and plant studies vs. hybridization under other approaches, and the use of population genetic vs. phylogeographic studies. Period surveyed: January 1, 2000 to October 20, 2022, survey performed October 20, 2022.

Search Topics	Hits	% hits when 'plants' added to the search	% hits with 'Phylogeography' vs. 'Population genetics'	% hits with 'hybrid zone' vs. 'hybridization'	URL of the search
hybrid zone' AND 'population genetics'	1622			8.48	https://www.webofscience.com/wos/alldb/summary/80fd5f8b-1475-4e99-8e81-f63bf794d80b-578d5037/relevance/1
hybrid zone' AND 'phylogeography'	336		20.72	15.19	https://www.webofscience.com/wos/alldb/summary/87352f33-0ca8-4cf2-bc0f-6892c9babc60-578d9fb2/relevance/1
hybridization' AND 'population genetics'	19138				https://www.webofscience.com/wos/alldb/summary/1f08c0a6-1c5f-45fb-8f7f-41635a7ad49b-578da51a/relevance/1
hybridization' AND 'phylogeography'	2212		11.56		https://www.webofscience.com/wos/alldb/summary/a465cf3a-bc4c-465a-ae4d-9014a69bf54f-578dae82/relevance/1
hybrid zone' AND 'population genetics' AND 'plants'	319	19.67		4.41	https://www.webofscience.com/wos/alldb/summary/86181a43-6be1-4c8b-9841-2cfbcb9674fc-578dba58/relevance/1
hybrid zone' AND 'phylogeography' AND 'plants'	48	14.29	15.0	6.89	https://www.webofscience.com/wos/alldb/summary/017d6982-92ad-420f-9aa2-ad61429ff3c3-578dc385/relevance/1
hybridization' AND 'population genetics' AND 'plants'	7240	37.83			https://www.webofscience.com/wos/alldb/summary/8427f1e0-6dfe-4e25-9ad7-1d1cf5cfe632-578dcc3f/relevance/1
hybridization' AND 'phylogeography' AND 'plants'	697	31.51	9.63		https://www.webofscience.com/wos/alldb/summary/f38b4464-e955-41cd-9229-cf76fa5071bd-578dd067/relevance/1
		no plants>>plants	population genetics>>phylogeography	hybridization >> hybrid zone	
